# *p53* maintains lineage fidelity during lung capillary injury-repair in neonatal hyperoxia

**DOI:** 10.1172/jci.insight.182880

**Published:** 2025-08-05

**Authors:** Lisandra Vila Ellis, Jonathan D. Bywaters, Amanda Ceas, Yun Liu, Jennifer M.S. Sucre, Jichao Chen

**Affiliations:** 1 Department of Cell & Developmental Biology, Feinberg School of Medicine, Northwestern University, Chicago, Illinois, USA.; 2 Department of Pulmonary Medicine, University of Texas MD Anderson Cancer Center, Houston, Texas, USA.; 3 UT Health Science Center, San Antonio, Texas, USA.; 4 Department of Pediatrics, Vanderbilt University Medical Center, Nashville, Tennessee, USA.; 5 Department of Cell and Developmental Biology, Vanderbilt University, Nashville, Tennessee, USA.; 6 Biodevelopmental Origins of Lung Disease Center, Vanderbilt University Medical Center, Nashville, Tennessee, USA.; 7 Department of Pediatrics, Perinatal Institute, Division of Pulmonary Biology, University of Cincinnati and Cincinnati Children’s Hospital Medical Center, Cincinnati, Ohio, USA.

**Keywords:** Development, Pulmonology, Vascular biology, Endothelial cells, p53

## Abstract

Bronchopulmonary dysplasia (BPD), a prevalent and chronic lung disease affecting premature newborns, results in vascular rarefaction and alveolar simplification. Although the vasculature has been recognized as a main player in this disease, the recently found capillary heterogeneity and cellular dynamics of endothelial subpopulations in BPD remain unclear. Here, we showed that Cap2 cells were damaged during neonatal hyperoxic injury, leading to their replacement by Cap1 cells, which, in turn, significantly declined. Single-cell RNA-Seq identified the activation of numerous p53 target genes in endothelial cells (ECs), including *Cdkn1a* (*p21*). While global deletion of *p53* resulted in worsened vasculature, EC-specific deletion of *p53* reversed the vascular phenotype and improved alveolar simplification during hyperoxia. This recovery was associated with the emergence of a transitional EC state, enriched for oxidative stress response genes and growth factors. Notably, this transitional EC gene signature was conserved in an aberrant capillary population identified in human BPD with pulmonary hypertension, underscoring the biological and clinical relevance of our findings. These results reveal a key role for *p53* in maintaining endothelial lineage fidelity during pulmonary capillary repair following hyperoxic injury and highlight the critical contribution of the endothelium to BPD pathogenesis.

## Abstract 2

Premature babies with bronchopulmonary dysplasia suffer from lung damage. Our study shows how vascular cells and genes play a role in repairing this damage, offering potential treatments.

## Graphical abstract

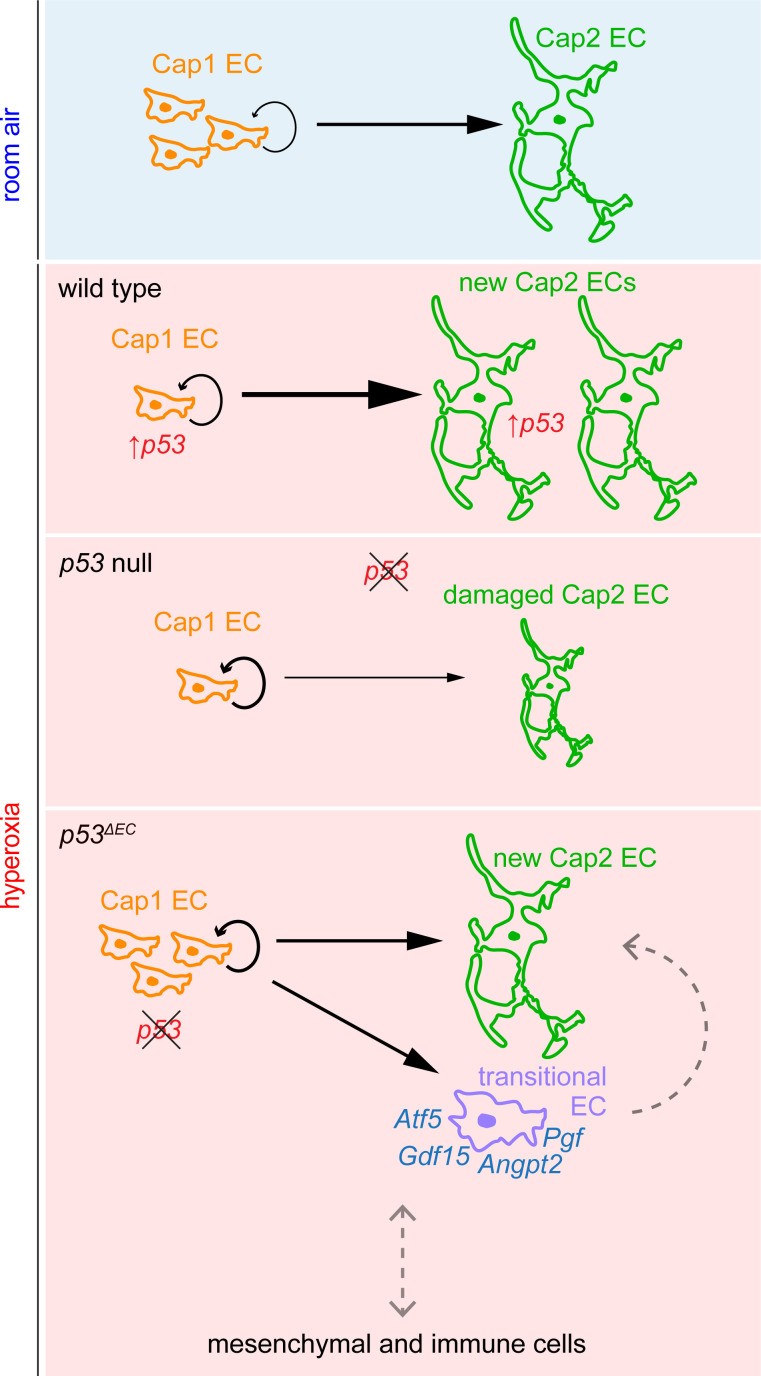

## Introduction

Bronchopulmonary dysplasia (BPD) is a chronic disease that develops in premature newborns following ventilation and oxygen treatment for acute respiratory failure due to their shortened gestation (1). BPD results in disrupted lung development characterized by alveolar simplification (enlarged alveoli with reduced septation) and vascular rarefaction (reduced and dysmorphic vasculature) (1). Historically, much of the focus has been on the epithelium and mesenchyme as the target cells that underlie BPD pathology (2). However, limited studies propose the vasculature as a main player in the pathogenesis of BPD, something known as the “vascular hypothesis” (1). Recent studies have explored the transcriptomic changes in mouse models of BPD using single-cell RNA sequencing (scRNA-Seq) (3–5); however, the endothelium-specific contributions remain unexplored.

Using scRNA-Seq, we and others have shown that lung capillaries are made up of 2 transcriptionally distinct endothelial cell (EC) populations — PLVAP^+^ ECs (also known as Cap1 or gCap) and CAR4^+^ ECs (Cap2, aCap, or aerocytes) (6–8). These 2 populations also differ in morphology and localization within the alveolar space, with Cap2 cells being web like, larger, and contacting the epithelium without intervening cells. Cap1 cells, on the other hand, account for most of the capillary cells, have a conventional EC morphology, and do not directly contact the epithelium (6). Alveolar type 1 (AT1) cells, their ultrathin epithelial counterpart in the air-blood barrier, secrete vascular endothelial growth factor A (*Vegfa*), a signal required for Cap2 cell specification (6). Additionally, our work revealed that Cap2 cells are located in regions undergoing secondary septation and aid in AT1 cell folding during alveologenesis (6, 9). This critical step of alveologenesis is compromised in BPD (1), suggesting the endothelium could be a major contributor to this disease.

In this study of the endothelium-specific response in BPD, we employed a widely used mouse model of neonatal hyperoxia coupled with EC type–specific drivers, which allowed us to investigate endothelial injury. scRNA-Seq analysis revealed endothelial upregulation of Trp53 (p53) target genes in this model, prompting us to test the function of *p53* using both a global knockout and an EC-specific deletion. Strikingly, endothelial *p53* deletion followed by hyperoxic injury lead to the emergence of a previously unknown transitional EC state, associated with improvements in both vascular and alveolar phenotypes.

## Results

### Neonatal hyperoxia results in decreased Cap1 vasculature.

**Figure 1:**
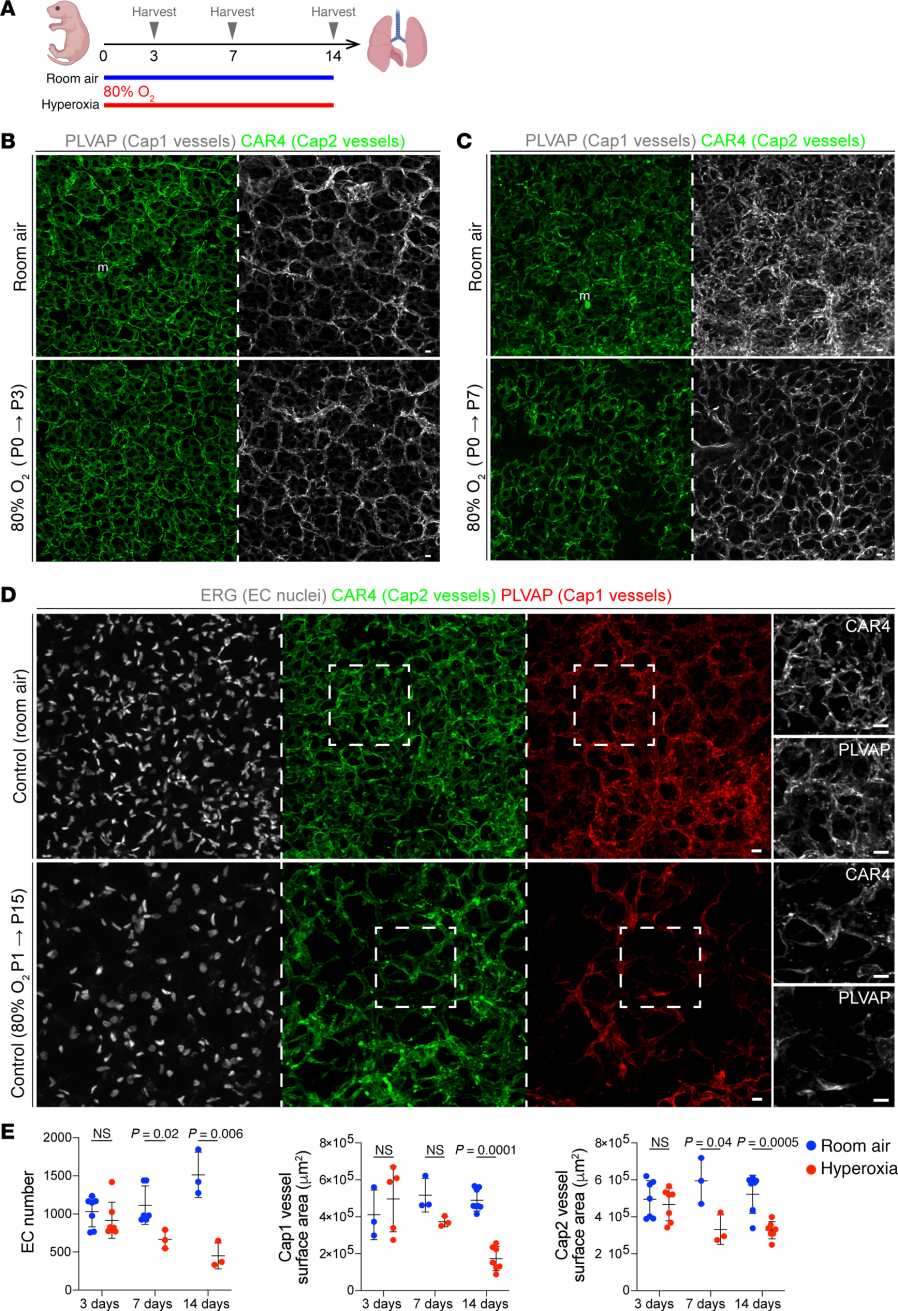
Hyperoxia exposure results in alveolar simplification with cell type–specific effects in ECs. (**A**) Experimental model showing continuous neonatal exposure to hyperoxia (80% O_2_) or room air for 3, 7, or 14 days. Created with BioRender.com. (**B** and **C**) En face view of immunostained lungs showing the effect of 3 days (**B**) and 7 days (**C**) of hyperoxia exposure on Cap1 vasculature (PLVAP) and Cap2 vasculature (CAR4) compared with room air control. (**D**) En face view of immunostained lungs showing the effect of 14 days of hyperoxia on EC number (ERG), Cap1 vasculature (PLVAP), and Cap2 vasculature (CAR4) compared with room air control. Boxed regions are shown at higher magnification to the right. (**E**) Quantifications show that hyperoxia results in a significant reduction in total EC number (ERG), Cap1 vasculature surface area (PLVAP), and Cap2 vasculature surface area (CAR4), with the most dramatic reduction being Cap1 specific (Student’s *t* test). Images are representative of at least 3 littermate pairs. For quantification, each symbol represents the average of 3 distinct regions imaged within 1 mouse lung. m, macrophage. Scale bars: 10 μm.

To investigate how neonatal hyperoxia disrupts the lung vasculature, we exposed newborn mouse pups to either hyperoxia (80% O_2_) or room air (21% O_2_) continuously for 14 days, without a recovery period (Figure 1A). Although multiple hyperoxia protocols were considered, prolonged exposure to 80% O_2_ is an established model in which injury predominates and has been shown to more robustly reproduce the blunting of secondary septation and increased septal wall thickness observed in patients with BPD (10–12). We evaluated epithelial and vascular integrity and found that compared with those in room air, mice in hyperoxia showed reduced vasculature as expected and an enlargement of the airspace (Supplemental Figure 1, A and B; supplemental material available online with this article; https://doi.org/10.1172/jci.insight.182880DS1).

To better understand the timing of Cap1/Cap2-specific injury in this model, we conducted immunostaining at various time points, including 3, 7, and 14 days after hyperoxia (Figure 1, A–D). After 3 days of hyperoxia, EC number, Cap1, and Cap2 vessel area remained unchanged (Figure 1, B and E). By day 7, a reduction in EC number and Cap2 vessel area became noticeable, suggesting that Cap2 cells are initially affected and possibly more susceptible to hyperoxia (Figure 1, C and E). At that time, Cap1 vessels also appear to be impacted but no significant difference was detected (Figure 1, C and E). By day 14, we observed an overall reduction in EC number and total vessel area, accompanied by a preferential loss of Cap1 cell expression of its marker gene during hyperoxia, representing a decline in Cap1 vasculature (Figure 1, D and E). Conversely, CAR4 expression remained uniformly present in the remaining vasculature, in contrast to its patchy loss after 7 days of injury (Figure 1, C and D). In addition, the macrovasculature seemed unaffected at a morphological level (Supplemental Figure 1C). These findings suggest EC type–specific damage and response to hyperoxia, mainly arising from the capillaries.

In patients with BPD, male newborns have shown worse outcomes compared with female newborns (13), and these sex disparities have been explored at the transcriptional level (3, 13, 14). Therefore, we compared Cap1 and Cap2 vessel areas in male and female mice across conditions after 14 days of hyperoxia but found no significant differences (Supplemental Figure 1D).

### Cap2 cells are damaged upon hyperoxia and replaced by Cap1 cells, exacerbating Cap1 cell loss.

To evaluate EC dynamics in this BPD model, we exposed pups prelabeled with Cap1- or Cap2-specific drivers to hyperoxia. Using scRNA-Seq, we identified KIT proto-oncogene receptor tyrosine kinase (*Kit*) as a specific marker for Cap1 cells and carbonic anhydrase 4 (*Car4*) as a marker for Cap2 cells (Supplemental Figure 2A). We obtained a *Kit^CreERT2^* mouse and tested its recombination efficiency and specificity by crossing it to a *Rosa^Sun1GFP^* reporter (15, 16). Injection of postnatal pups with tamoxifen for 24 hours revealed it to be highly efficient (92%) and specific (100%) (Supplemental Figure 2, B and C). We then coupled this driver with the reporter *Rosa^tdT^* for lineage tracing of Cap1 cells (17). For Cap2 cells, lineage tracing was accomplished using our recently developed *Car4^CreER^* mouse (18). To maximize cell recombination, a high dose of tamoxifen was administered. We implemented a 48-hour interval before hyperoxia exposure to allow sufficient time for tamoxifen clearance to (a) minimize injury-induced, nonspecific driver induction and labeling and (b) mitigate heightened mortality risk during hyperoxia posttamoxifen injection (19) (Supplemental Figure 2D).

**Figure 2:**
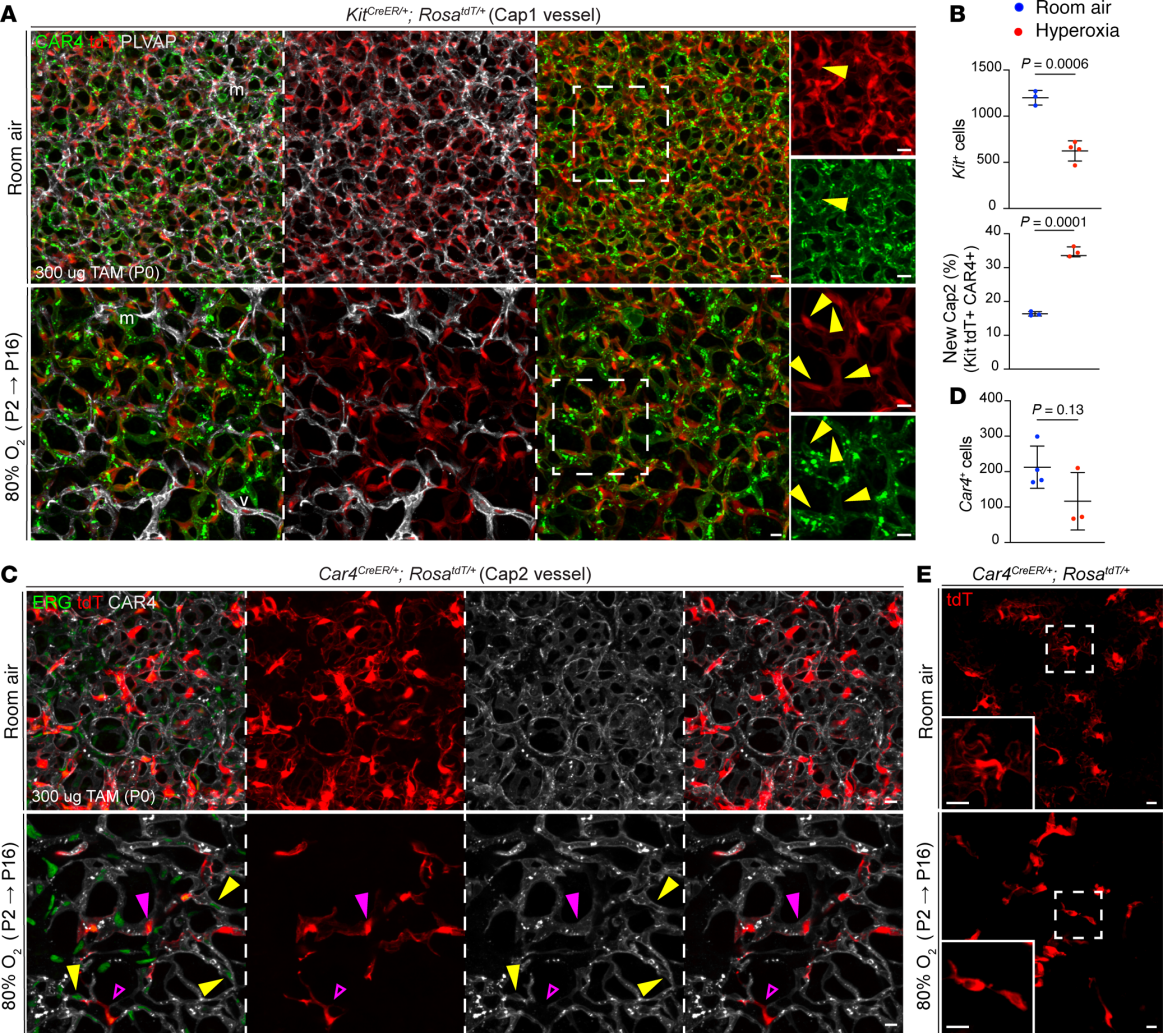
Cap1 ECs convert to Cap2 to replace cell type–specific EC loss during hyperoxia. (**A**) En face view of immunostained lungs from lineage-traced *Kit^CreER^; Rosa^tdT/+^* mice, demonstrating a decrease in Cap1 EC number in hyperoxia relative to room air. Hyperoxia-treated lungs also exhibited a stark increase in the proportion of tdT^+^ cells that express CAR4 (yellow arrowheads), suggesting increased conversion from Cap1 to Cap2 in hyperoxia. Boxed regions are shown at a higher magnification to the right. (**B**) Top: Quantification of tdT^+^ Cap1 ECs in room air and hyperoxia, revealing a significant decrease in lineage-traced Cap1 ECs (Student’s *t* test). Bottom: Quantification of the proportion of Car4^+^tdT^+^ cells of total tdT^+^ cells in room air and hyperoxia, confirming a significant increase in conversion from lineage-traced Cap1 ECs to Cap2 in hyperoxia (Student’s *t* test). (**C**) En face view of immunostained lungs from lineage-traced *Car4^CreER^; Rosa^tdT/+^* mice. Hyperoxia exposure resulted in a reduction in tdT^+^ Cap2 cells, representing Cap2 ECs present before treatment (magenta arrowheads). Some remaining tdT^+^ cells showed a loss in CAR4 expression in hyperoxia (open arrowheads), while tdT^–^ Cap2 cells (CAR4^+^) also appeared in hyperoxia, mostly representing new Cap2 cells converted from Cap1 (yellow arrowheads). (**D**) Quantification of tdT^+^ Cap2 ECs in room air and hyperoxia, showing a downward trend in lineage-traced Cap2 ECs (Student’s *t* test). (**E**) En face view of immunostained lungs showing individual tdT-labeled Cap2 cells in room air and hyperoxia, demonstrating the loss of the expansive, net-like morphology of Cap2 cells upon sustained hyperoxic injury. Boxed regions are shown at a higher magnification in insets. Images are representative of at least 3 littermate pairs. For quantification, each symbol represents the average of 3 distinct regions imaged within 1 mouse lung. m, macrophage; v, large vessel. TAM, 300 μg tamoxifen administered at P0. Scale bars: 10 μm.

Given the substantial accumulation of the reporter in the cell nucleus, we employed tdT to quantify individual cells, simultaneously matching the nucleus with PLVAP and CAR4 antibodies as surface markers. Lineage tracing of Cap1 cells confirmed a decrease in Cap1 cell number after 14 days of hyperoxia (Figure 2, A and B), which, although not significant, was also noticeable after 7 days of hyperoxia (Supplemental Figure 2E). Although some tdT^+^PLVAP^+^ Cap1 cells remained, we noticed that many Cap1 cells, identified by Kit tdT expression, now expressed CAR4, indicating an increase in transition from Cap1 to Cap2 cells (Figure 2, A and B). We previously showed that both Cap1 and Cap2 cells arise from endothelial progenitors that closely resemble Cap1 cells and express *Kit* (6), and this transition was corroborated by others (7). Additionally, Cap1 cells have been proposed to act as a source of new Cap2 cells upon injury and regeneration (8). While around 15% of Cap1 cells were observed transitioning to Cap2 fate in room air, which is expected at this early developmental stage, we observed a 2-fold increase in Cap1-to-Cap2 conversion in hyperoxia (Figure 2, A and B).

This transition from Cap1 to Cap2 cells, in combination with the decrease in CAR4 expression after 7 days of hyperoxia (Figure 1C), led us to hypothesize that Cap2 cells are susceptible to hyperoxic injury. Employing lineage tracing to label Cap2 cells before exposure to hyperoxia, we noted a substantial reduction in the number of tdT^+^ cells, representing original Cap2 cells (preinjury), despite the emergence of CAR4^+^tdT^–^ cells, signifying new Cap2 cells converted from Cap1 cells (Figure 2, C and D). The loss of Cap2 cells was confirmed after 7 days of hyperoxia treatment (Supplemental Figure 2F), and immunostaining with cleaved-CASP3 to assess apoptosis confirmed that originally labeled Cap2 cells experienced death at a higher rate than room air (Supplemental Figure 2G). Additionally, some tdT^+^ cells in hyperoxia exhibited loss of CAR4 expression, suggesting that injured Cap2 cells could lose their marker genes predating cell death. Our previous work highlighted the expansive morphology of Cap2 cells, allowing them to contribute to multiple vessel segments (6). We used the same model of sparse cell labeling to visualize individual Cap2 cell morphology. We observed that, in hyperoxia, Cap2 cells deviated from their typical web-like morphology, displaying a range of shapes but consistently losing their cellular projections (Figure 2E), possibly due to cell shrinkage prior to death (Supplemental Figure 2G).

To summarize the cellular changes, we observed that upon hyperoxia treatment (a) both Cap1 and Cap2 cells were damaged and decreased in number; (b) surviving Cap2 cells lost their distinct morphology and markers; and (c) Cap1 cells transitioned into Cap2 cells. During normal development in room air, Cap1 cells proliferate and undergo limited conversion into Cap2 cells (6). In hyperoxia, however, Cap1 cells convert at a high rate with a net result of greater impact on Cap1 than Cap2 cells.

### Neonatal hyperoxia upregulates p53 target genes.

**Figure 3:**
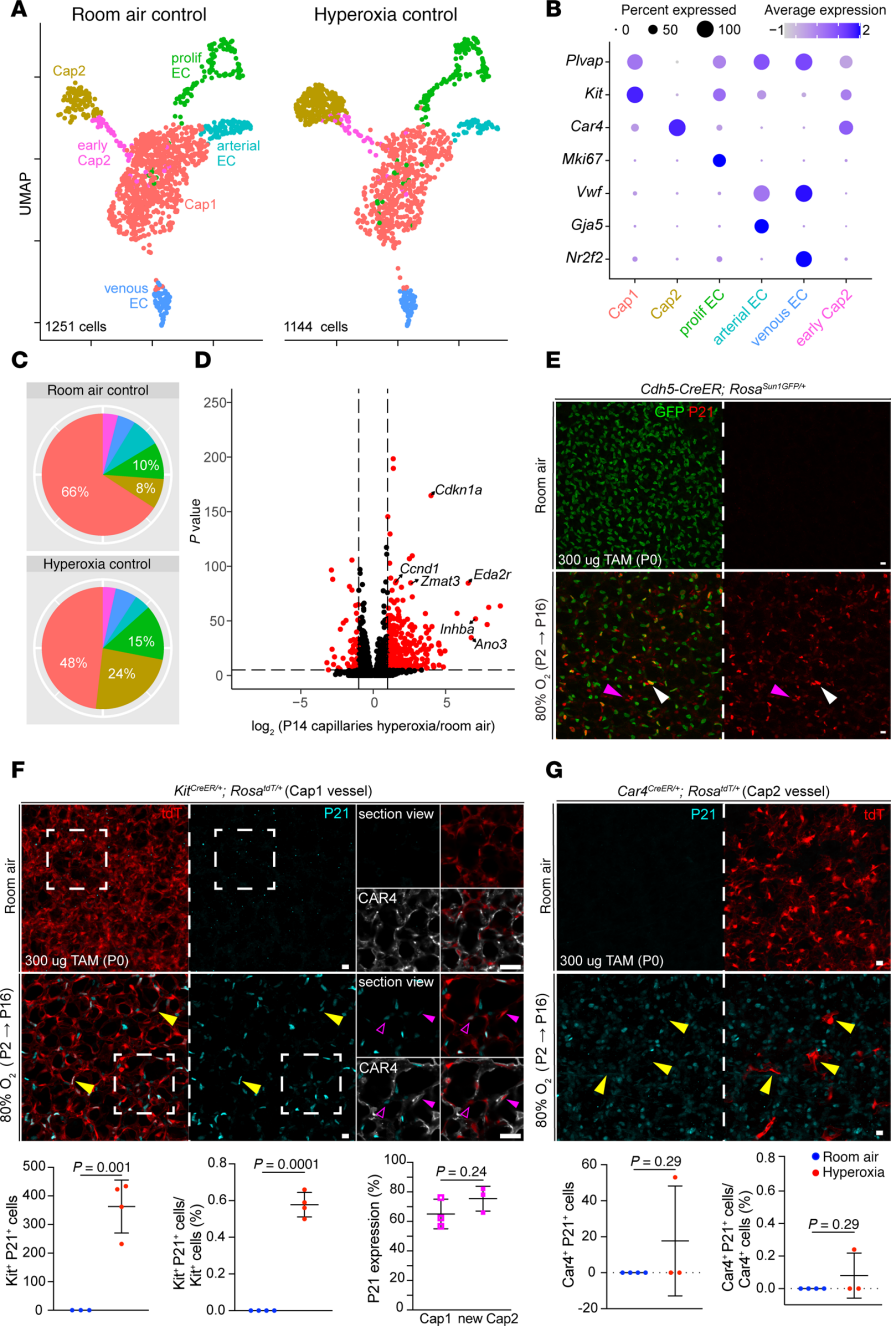
Hyperoxia exposure causes upregulation of *p53* target genes in capillary ECs. (**A**) UMAP of purified lung ECs in room air and hyperoxia. ECs are color coded according to cell population. (**B**) Dot plot showing markers used to identify each EC population. (**C**) Pie charts demonstrating the proportions of each EC population in both conditions. Hyperoxia exposure results in a substantial reduction in Cap1 cells and an increase in Cap2 cells. (**D**) Volcano plot showing differential gene expression in capillaries between hyperoxia and room air lungs. Hyperoxia exposure causes significant upregulation of many p53 target genes in capillary ECs. (**E**) En face view of immunostained lungs showing GFP-labeled ECs in room air and hyperoxia. Hyperoxic injury causes widespread upregulation of *p53* target gene P21 in ECs (white arrowheads) and non-ECs (magenta arrowheads) compared with room air control. (**F**) En face view of immunostained lungs showing nearly 60% of lineage-traced (tdT^+^) Cap1 cells express P21 in hyperoxia (yellow arrowheads) and its associated quantification (below; Student’s *t* test). Boxed regions are shown at higher magnification and shown as a section view. Both CAR4^–^tdT^+^ Cap1 cells (representing nonconverted Cap1 ECs; open magenta arrowheads) and CAR4^+^tdT^+^ Cap2 cells (representing Cap1 cells converted into Cap2; magenta arrowheads) were found to express P21 with no enrichment observed in either population. (**G**) En face view of immunostained lungs showing most lineage-traced Cap2 cells were found to lack P21 expression in hyperoxia (yellow arrowheads). Images are representative of at least 3 littermate pairs. For quantification, each symbol represents the average of 3 distinct regions imaged within 1 mouse lung. TAM, 300 μg tamoxifen administered at P0. Scale bars: 10 μm.

To examine the transcriptional changes of ECs in hyperoxia, we performed scRNA-Seq after 14 days of 80% O_2_ exposure and assessed a total of 2,395 sorted ECs (Figure 3A). Using established marker genes (6), each endothelial population was identified (Figure 3B). In accordance with our previous work (6), in room air Cap1 cells represented the bulk of the lung vasculature, while Cap2 cells were a small but distinct cluster (Figure 3C and Supplemental Table 1). However, in hyperoxia, scRNA-Seq revealed a decrease in Cap1 cells and a proportional increase in Cap2 cells (Figure 3C). These changes were consistent with our immunostaining and lineage-tracing observations, including the conversion from Cap1 to Cap2 fate (Figure 1D, Figure 2A, and Supplemental Figure 3A). Notably, proliferative ECs also increased in number under hyperoxia (Figure 3C).

We analyzed gene expression across the entire capillary population under both conditions and found a significant upregulation of p53 target genes in hyperoxia, including *Cdkn1a* (*p21*), a cell cycle inhibitor that protects the lung from oxidative stress (20), as well as *Ano3*, *Ccnd1*, *Eda2r*, *Inhba*, and *Zmat3* (Figure 3D, and Supplemental Table 2). Importantly, this pattern of upregulation was conserved across capillary subtypes (Supplemental Figure 3B). A pathway analysis of the top upregulated genes in all the capillary ECs in hyperoxia and room air also identified the p53 pathway as highly enriched (Supplemental Figure 3C), and Western blot of P53 and phosphorylated P53 showed increased expression after 3 days of hyperoxia exposure (Supplemental Figure 3D). While *p53* activation in the hyperoxic lung has been documented before (21, 22), a recent publication investigating the p53/p21 pathway in hyperoxia was limited to Von Willebrand factor–positive (VWF^+^) macrovasculature, thus excluding the capillary response (23). To specifically label and trace ECs during hyperoxia, and because our EC nuclear marker (ERG) and P21 antibody were from the same species, we used a *Cdh5-CreER* mouse, a pan-endothelial driver, in combination with a *Rosa^Sun1GFP^* reporter (16). Immunofluorescence confirmed the P21 upregulation in ECs as well as non-ECs (Figure 3E). We also observed this upregulation by RNAscope (Supplemental Figure 3E). Further analysis of the scRNA-Seq data demonstrated *Cdkn1a* (*p21*) upregulation in all EC types in hyperoxia compared with room air, except arteries (Supplemental Figure 3F).

To better understand capillary-specific *p53* activation, we assessed the expression of P21 in the Cap1 and Cap2 lines, *Kit^CreER^* and *Car4^CreER^*, respectively, after hyperoxia (Figure 3, F and G). Approximately 60% of Kit^+^ cells (tdT^+^) demonstrated colocalization with P21, at a comparable frequency in Cap1 cells (tdT^+^CAR4^–^) and newly formed Cap2 cells (tdT^+^CAR4^+^) (Figure 3F). The extent of hyperoxia-induced alveolar and vascular simplification in this model varies among mice. In samples exhibiting more severe injury, as assessed by level of alveolar simplification, none of the initially labeled Cap2 cells expressed P21 (Figure 3G). In those with milder injury, more lineage-traced (tdT^+^) Cap2 cells persisted and few, but some, of these cells expressed P21.

Based on existing literature suggesting that hyperoxic injury increases cellular senescence in the postnatal lung, we considered the possibility that *p53* could be modulating this process in the ECs (23). Although *p21* and *p16* are both known to play a role in senescence, it is generally believed that *p21* is the main mediator (23). Our scRNA-Seq data show increased expression of *Cdkn1a* (*p21*), but not *Cdkn2a* (*p16*)*,* in hyperoxia compared with room air (Supplemental Figure 4A), suggesting that *p53* is not activating *p16* in this context. We then analyzed this scRNA-Seq data to generate gene scores using the Mayo senescence (senMayo) and senescence-associated secretory phenotype (SASP) gene lists and found a negligible increase in hyperoxia (Supplemental Figure 4B) (24). Activation of the p53 pathway can contribute to loss of LAMINB1, which is also associated with senescence (25). To further confirm whether capillary ECs are becoming senescent in hyperoxia, we performed immunofluorescence staining for LAMINB1 in our cell-type-specific mouse lines using either *Kit^CreERT2^* and *Car4^CreER^* exposed to hyperoxia for 14 days. Although loss of LAMINB1 is a hallmark of senescent cells, it was retained in both Cap1 and Cap2 vessels after 14 days of hyperoxia (Supplemental Figure 4, C and D). These findings do not suggest that *p53* is promoting a senescence phenotype. A possible explanation for this could involve timing, as senescence is a dynamic process that varies across developmental stages in both humans and mice (23). Arguably, since capillaries must react and adapt quickly to their environment, they may be less susceptible to this phenomenon. Alternatively, *p53* could be playing a different role involving lineage maintenance during Cap1 to Cap2 conversion, similar to its function in maintaining epithelial cell identity during lung injury and repair (26–28).

### Global deletion of p53 worsens Cap2 vasculature in hyperoxia.

**Figure 4:**
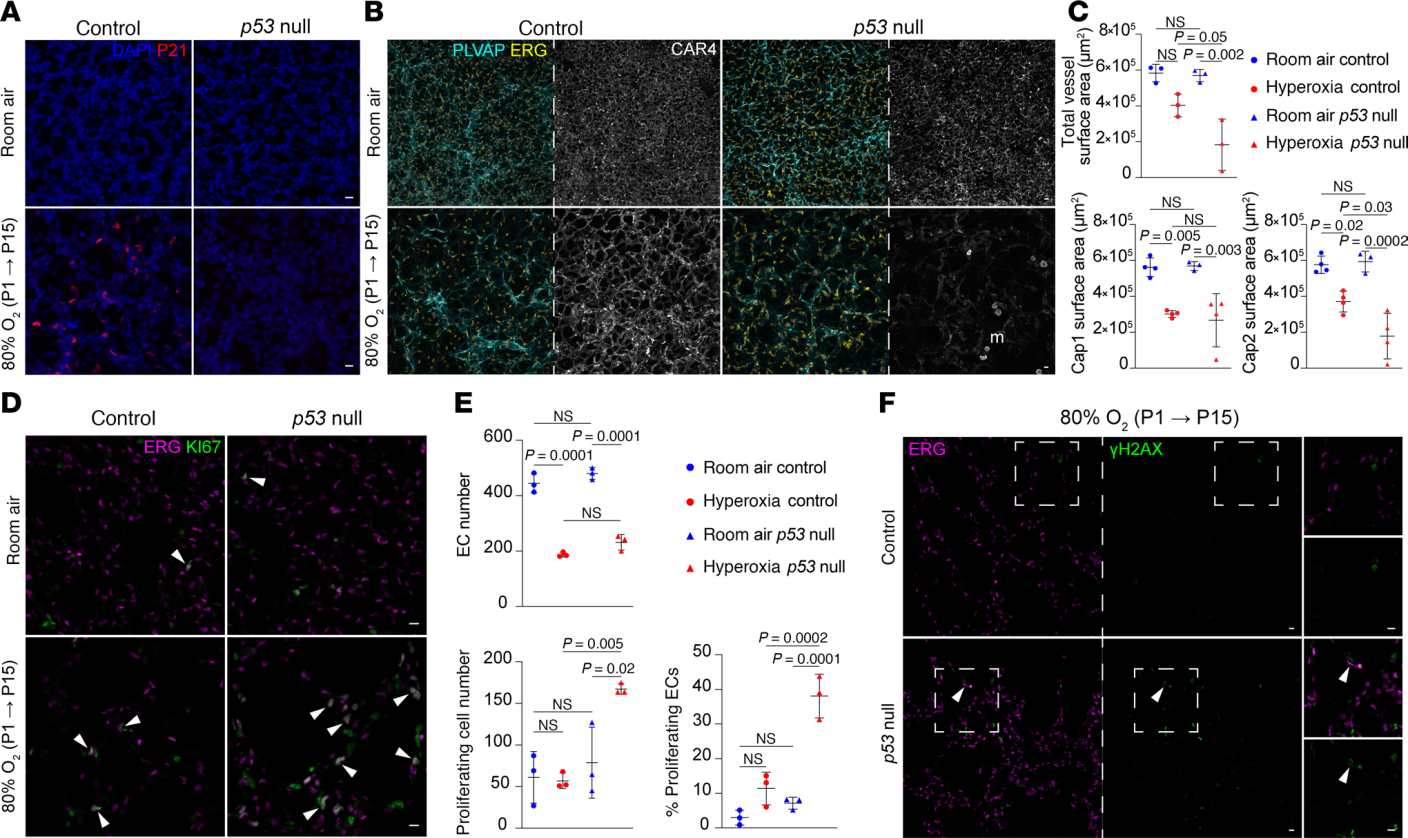
Global *p53* deletion in hyperoxia results in increased EC proliferation and Cap2-specific decrease. (**A**) En face view of immunostained lungs demonstrating efficient deletion of *p53* in the hyperoxia *p53-*null condition compared with hyperoxia control by P21 staining. (**B**) En face view of immunostained lungs showing the effect of *p53* deletion on total EC nuclei (ERG), Cap1 vasculature (PLVAP), and Cap2 vasculature (CAR4) in room air and hyperoxia, demonstrating a decrease in CAR4 staining in the hyperoxia *p53-*null group. (**C**) Quantification of Cap1 surface area, Cap2 surface area, and total vessel surface area (as measured by ICAM2 staining) in each condition (1-way ANOVA with Tukey’s multiple comparisons), demonstrating a significant reduction in Cap2 and total vessel area in the hyperoxia *p53-*null group. (**D**) Section immunostaining showing the effect of *p53* deletion and hyperoxia treatment on cell proliferation (KI67) in ECs (ERG, white arrowheads) and non-ECs. (**E**) Quantification showing hyperoxia exposure or *p53* deletion alone did not result in a significant increase in overall proliferation or EC proliferation compared with room air controls, but hyperoxia *p53-*null mice exhibited significant increases in both EC-specific and overall proliferation (1-way ANOVA with Tukey’s multiple comparisons). (**F**) Section immunostaining evaluating DNA damage (γH2AX) in hyperoxia-treated control and *p53-*null lungs. DNA damage is higher in hyperoxia *p53-*null lungs compared with hyperoxia controls, with sporadic colocalization of γH2AX to ECs (ERG) in the hyperoxia *p53-*null lung, indicating endothelial damage (white arrowheads). Boxed regions are shown at a higher magnification to the right. Images are representative of at least 3 littermate pairs. For quantification, each symbol represents the average of 3 distinct regions imaged within 1 mouse lung. m, macrophage. Scale bars: 10 μm.

To investigate the role of p53 pathway activation in hyperoxia, we obtained a *p53^flox^* mouse and crossed it with a *CMV-Cre* mouse (29, 30), inducing the deletion of the *loxP*-flanked gene in all tissues. We then selectively bred progeny lacking both the *flox* allele and *Cre*, establishing a *p53-*null mouse model. We confirmed the deletion of *p53* by immunostaining of its target gene P21 (Figure 4A). P21 expression was absent in room air (Figure 3, E–G, and Figure 4A), whereas it was evident in the control hyperoxia group. In the *p53-*null group, however, P21 was not detected, indicating robust deletion of *p53* (Figure 4A).

Assessment of the vascular phenotype revealed no differences in Cap1 and Cap2 cells between the control and p53-null mice in room air which was expected since we did not see P21 expression in room air (Figure 4, A and B). In hyperoxia, the *p53*-null mice exhibited a further decrease in CAR4 expression and thus Cap2 vessel area (Figure 4, B and C). These results could mean an increase in Cap2 cell death, damaged Cap2 cells with less marker expression, or decreased conversion from Cap1 to Cap2 fate. To further characterize the alveolar simplification phenotype normally observed in hyperoxia, we examined the alveolar surface of the lungs (Supplemental Figure 5A). Our analysis revealed that both control and *p53*-null mice in hyperoxia experience similar enlargement of airspace. Quantification of the airspace by mean linear intercept (MLI) did not find a significant difference between control and mutant in hyperoxia (Supplemental Figure 5A).

To dissect the cellular processes influenced by *p53* in hyperoxia within the vasculature, we examined endothelial proliferation in *p53*-null mice. In room air, consistent with the early developmental stage of the lungs, we observed a modest percentage of proliferative ECs, which did not significantly change in hyperoxia (Figure 4, D and E). However, in hyperoxia-treated *p53*-null mice, endothelial proliferation increased significantly (Figure 4, D and E). This increase may reflect ECs escaping DNA damage checkpoints and apoptosis, unrelated to conversion, as *p53* is known to respond to DNA damage (31). We assessed DNA damage using phosphorylated histone H2AX (γH2AX). In hyperoxia controls, rare γH2AX expression in non-ECs suggested effective repair. In contrast, hyperoxia-treated *p53*-null mice showed frequent γH2AX expression, with limited colocalization in ECs, implicating *p53* in DNA repair regulation in non-ECs during hyperoxia (Figure 4F). Because *p53* also mediates apoptosis in response to irreparable damage (28), we examined cleaved CASP3 expression (Supplemental Figure 5B). Although cell death increased in mutant mice, γH2AX and cleaved-CASP3 did not colocalize, suggesting sequential DNA damage and apoptosis. These findings suggest that without *p53*, Cap1 cells may preferentially proliferate rather than convert to Cap2 fate, contributing to reduced Cap2 vessels. The scarcity of γH2AX in ECs could reflect greater resilience, more rapid clearance, or faster repair following hyperoxia. To identify non-ECs with increased γH2AX in the hyperoxia *p53*-null lungs, we performed immunostaining for immune (CD45), epithelial (NKX2.1), and mesenchymal (IL33, MEOX2, PDGFRα, PDGFRβ) markers (Supplemental Figure 5, C–F). γH2AX did not colocalize with CD45 or NKX2.1 (Supplemental Figure 5, C and D), indicating that immune and epithelial cells were not affected. Notably, γH2AX colocalized with IL33 and MEOX2, markers of distal and proximal interstitial fibroblasts (32), but rarely with PDGFRα or PDGFRβ (Supplemental Figure 5, E and F), suggesting that DNA damage may occur in specific mesenchymal subsets. However, decreased PDGFRα expression in hyperoxia-treated *p53*-null mice may also explain the lack of observed damage in PDGFRα^+^ cells.

**Figure 5:**
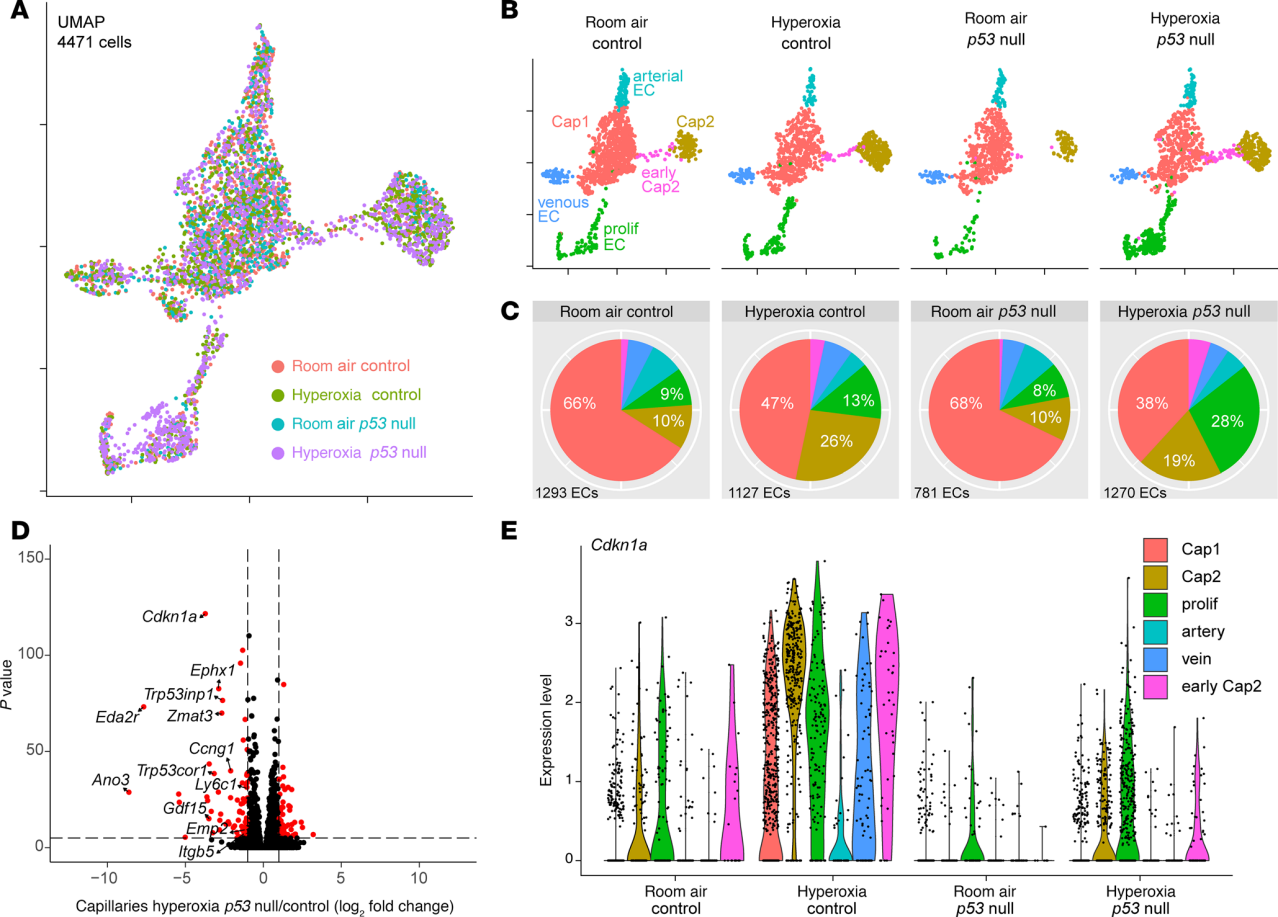
scRNA-Seq reveals transcriptional changes associated with deletion of *p53* in hyperoxia-treated *p53-*null lung ECs. (**A**) UMAP of lung ECs, color coded and overlaid from 4 different experimental conditions. (**B**) UMAPs of lung ECs from each experimental condition, color coded according to cell population. (**C**) Pie charts showing the proportion of each EC population in each experimental condition. Notably, proliferative ECs represent a larger proportion of ECs in the hyperoxia *p53-*null lung compared with any other condition. (**D**) Volcano plot depicting differentially expressed genes in capillaries between the hyperoxia *p53-*null versus the hyperoxia control conditions. (**E**) Violin plots showing expression level of p53 target *Cdkn1a* in each experimental condition, split by EC population. As compared with the hyperoxia control condition, hyperoxia *p53-*null lung ECs exhibit drastically reduced *Cdkn1a* expression across all populations.

To dissect the transcriptomic changes in the *p53-*null group, we conducted scRNA-Seq, profiling 4,471 ECs across control and mutant mice in both room air and hyperoxia (Figure 5A). Identification of all endothelial subpopulations was achieved using previously described markers (Figure 3B and Figure 5B), and the proportions of each cell type were calculated (Figure 5C and Supplemental Table 1). Consistent with immunostaining results, proliferative ECs substantially increased in the hyperoxia-treated *p53-*null group. Surprisingly, the *p53-*null Cap2 cluster in hyperoxia was larger than that in room air, albeit smaller than that in the hyperoxia control (Figure 5C). It is possible that Cap2 cells are present but lack expression of their mature markers such as CAR4 (Figure 4B), making them detectable by scRNA-Seq but not by staining. Additionally, smaller, more damaged, or less mature Cap2 cells may be disproportionally captured during sorting. To test this, we created a list of Cap2 markers expressed in room air at P14 and used it to create a gene score to evaluate the changes in Cap2 cells across different conditions (Supplemental Figure 6A). This revealed that Cap2 cells in the *p53-*null group in hyperoxia downregulate their marker genes (Supplemental Figure 6B). In a closer look, we compared the top 21 Cap2 genes across Cap2 ECs in all 4 samples and saw a distinct downregulation in the *p53-*null group in hyperoxia, confirming these cells are more damaged (Supplemental Figure 6C). Differential gene analysis comparing control and *p53-*null mice in hyperoxia revealed the expected changes associated with the deletion of *p53*, with a decline in p53 target genes, such as *p21* (*Cdkn1a*), as well as some Cap2 marker genes such as *Emp2* and *Itgb5* (Figure 5, D and E, and Supplemental Table 3). Altogether, as evidenced by a reduction in Cap2 vasculature and loss of Cap2 mature marker genes, it seems that *p53* protects conversion from Cap1 to Cap2 cell fate in neonatal hyperoxia.

### Conditional deletion of p53 in the endothelium improves vascular and alveolar simplification in hyperoxia.

**Figure 6:**
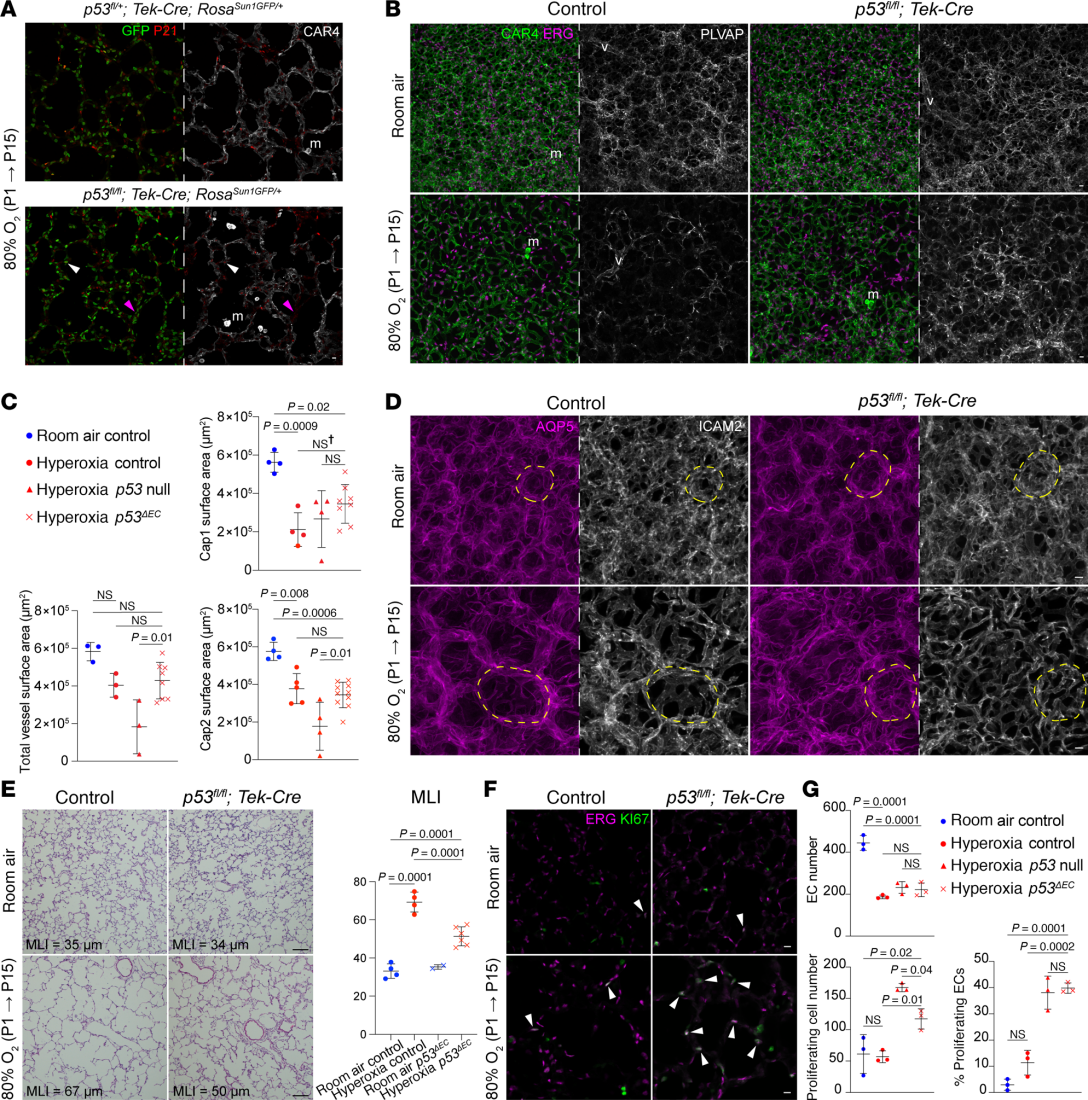
Endothelium-specific *p53* deletion in hyperoxia results in partial rescue of BPD phenotype. (**A**) Immunostained lung sections showing efficient endothelial deletion of *p53* in ECs using a *Tek-Cre* driver and *Rosa^Sun1GFP^*, as measured by P21 and GFP. Mutant lungs retained occasional P21 expressing escapers in both Cap1 (magenta arrowheads) and Cap2 ECs (white arrowheads). (**B**) En face view of immunostained lungs demonstrating the effect of EC-specific *p53* deletion in room air and hyperoxia on EC number (ERG), Cap1 vessels (PLVAP), and Cap2 vessels (CAR4). *p53*^ΔEC^ lungs demonstrate a substantial improvement in Cap1 vasculature in hyperoxia compared with control. (**C**) Quantification showing Cap1 vessel area is increased in the hyperoxia *p53*^ΔEC^ condition compared with the hyperoxia control condition, while Cap2 area is relatively unchanged. Compared with the hyperoxia *p53-*null lung, the hyperoxia *p53*^ΔEC^ lung shows a significant rescue of Cap2 area and total vessel area (1-way ANOVA with Tukey’s multiple comparisons). (**D**) En face view of immunostained lungs demonstrating alveolar islands (circled regions) with improved overall vasculature (ICAM2) in *p53*^ΔEC^ hyperoxia, along with a reduction in alveolar simplification (AQP5). (**E**) H&E-stained lung sections from each experimental condition and MLI quantification showing a significant reduction in alveolar simplification in the hyperoxia *p53*^ΔEC^ (1-way ANOVA with Tukey’s multiple comparisons). (**F**) Immunostained lung sections showing increased proliferation (KI67) in hyperoxia *p53*^ΔEC^ lungs compared with controls, mostly in ECs (ERG, white arrowheads). (**G**) Quantification reveals no change in EC number in hyperoxia *p53*^ΔEC^ compared with hyperoxia controls, and comparable endothelial proliferation between *p53-*null and *p53*^ΔEC^ lungs (1-way ANOVA with Tukey’s multiple comparisons). For quantification, each symbol represents the average of 3 distinct regions imaged within 1 mouse lung. m, macrophage; v, large vessel. Scale bars: 10 μm (white bars); 100 μm (black bars). ^†^Significant as measured with Student’s *t* test.

To explore the endothelium-specific role of *p53*, we conditionally deleted it using the pan-endothelial driver *Cdh5-CreER* (33). Despite efficient *p53* deletion, as indicated by P21 staining, Cap1 cells exhibited an overall improvement (Supplemental Figure 7A). However, due to variability in recombination efficiency and increased mortality associated with the combination of tamoxifen injections and hyperoxia, we opted for the use of a *Tek-Cre* (or *Tie2-Cre*) mouse instead (34), hereafter referred to as *p53^ΔEC^*. While *Tek* is enriched postnatally in Cap1 cells, it is expressed embryonically in the endothelial progenitors that give rise to both Cap1 and Cap2 (Supplemental Figure 7B); thus, this model is expected to achieve pan-endothelial deletion. Additionally, lineage tracing with the *Rosa^tdT^* reporter mouse showed that we can target both Cap1 and Cap2 cells with this driver with few escapers (Supplemental Figure 7C). Once again, since the antibodies were the same species, we employed a *Rosa^Sun1GFP^* reporter to validate the deletion by colocalizing GFP and P21 staining. We observed that most recombined cells exhibited a loss of P21 expression, except for rare escapers, which typically coexpressed CAR4 (Figure 6A), and we also confirmed these findings via RNAscope (Supplemental Figure 7D).

As deletion was confirmed, we continued to analyze the EC type–specific phenotype comparing the *p53^ΔEC^* mice in room air and hyperoxia conditions (Figure 6B). Recapitulating the *Cdh5-CreER* model, the vasculature in the *p53^ΔEC^* hyperoxia group showed remarkable improvement compared with the hyperoxia control group (Figure 6B). Specifically, Cap1 vessel area showed an upward trend, suggesting a recovery in the hyperoxia-treated *p53^ΔEC^* mice compared with hyperoxia control mice, while Cap2 vessel area was maintained compared with hyperoxia controls (Figure 6C). We detected an increase in Cap2 vasculature and total vessel area comparing *p53-*null and *p53^ΔEC^* mice in hyperoxia (Figure 6C). Moreover, the alveolar region of the *p53^ΔEC^* mice in hyperoxia showed improvement in alveolar angiogenesis and folding of the AT1 cell surface when compared with the controls in hyperoxia (Figure 6D). To quantify changes in airspaces, we measured MLI and found a significant decrease in alveolar size, indicating a partial rescue in the alveolar simplification — a key feature in BPD (Figure 6E).

To explore whether these changes could be attributed to an upsurge in EC proliferation, we quantified EC number and percentage of proliferating ECs (Figure 6, F and G). Despite an increase in endothelial proliferation, there was no significant increase in EC number in the *p53^ΔEC^* mice in hyperoxia when compared with hyperoxia control or *p53-*null hyperoxia mice (Figure 6G). We then examined DNA damage between hyperoxia control and *p53^ΔEC^* mice but found few cells experiencing this process (Supplemental Figure 7E). Compared with the hyperoxia *p53*-null mice — which shows an increase in non-EC DNA damage (Figure 4F) — the endothelial deletion of *p53* in hyperoxia resulted in little γH2AX, indicating that in general, ECs are more resilient to hyperoxic injury than non-ECs.

Given the discrepancy between the *p53-*null and *p53^ΔEC^* mice — namely, the (a) reduced Cap2 vessel area in the former, (b) increased Cap1 vessel area in the latter, and (c) high non-EC γH2AX in the *p53*-null mice — we explored possible non-cell autonomous contributions explaining these differences. This analysis is particularly relevant since other cell types also upregulate p53 target genes in this context (Supplemental Figure 7F). Our previous work highlighted the interplay between the epithelium and the vasculature during development, where Cap2 cells are specified by AT1-specific VEGFA (6). Consequently, we considered the epithelium as a good candidate for this compensation and deleted *p53* using a pan-epithelial driver, *Shh^Cre^* (35) (Supplemental Figure 7G). However, the deletion of *p53* in the epithelium had no effect on the hyperoxia phenotype in the vasculature (Supplemental Figure 7G).

### Transitional ECs arise upon conditional deletion of p53 in the endothelium in hyperoxia.

**Figure 7:**
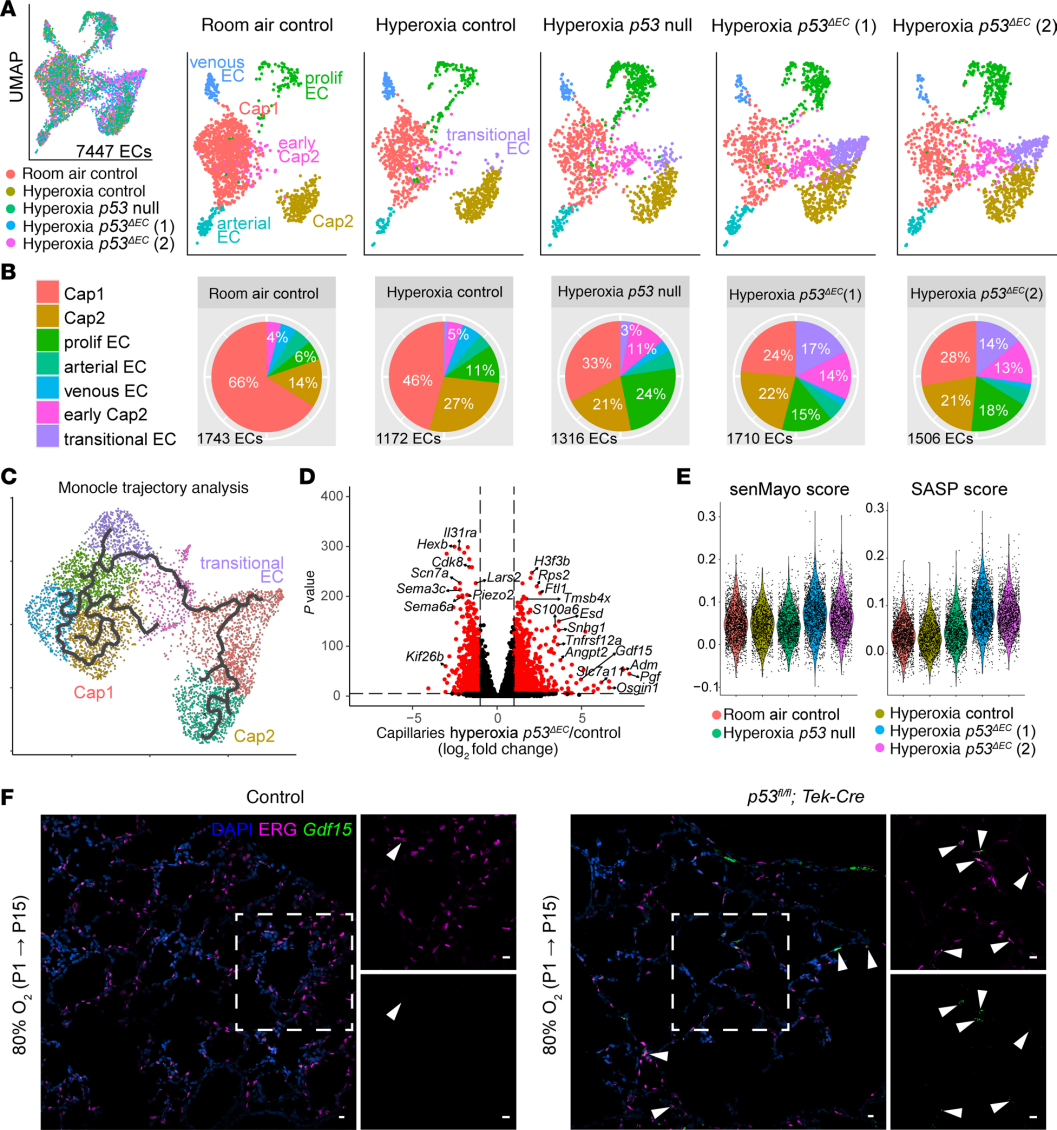
scRNA-Seq reveals a novel transitional EC population in the hyperoxia *p53^ΔEC^* lung. (**A**) UMAP of lung ECs, color coded and overlaid from each experimental condition (left) and then split by experimental condition and color coded according to EC population (right). Notably, the hyperoxia *p53*^ΔEC^ lungs demonstrate a potentially novel transitional EC cluster and an increased number of early Cap2 cells. (**B**) Pie charts showing the proportion of individual EC populations in each experimental condition. (**C**) Monocle trajectory analysis UMAP showing the intermediate transcriptional profile of the transitional EC cluster. (**D**) Volcano plot showing differentially expressed genes in the capillaries of the hyperoxia *p53*^ΔEC^ lung compared with the hyperoxia control, including the upregulation of *Pgf*, *Gdf15*, *Angpt2*, and *Osgin1*. (**E**) Violin plots featuring senMayo and SASP gene scores for each condition, which show an increase in the hyperoxia-treated *p53*^ΔEC^ lungs. (**F**) RNAscope in situ hybridization and immunostained lung sections showing upregulation of *Gdf15* in transitional ECs (white arrowheads) in the hyperoxia *p53*^ΔEC^ lung compared with the hyperoxia control. Scale bars: 10 μm.

To further explore these conflicting phenotypes, we conducted scRNA-Seq of 7447 ECs across the different conditions (Figure 7A). Since no differences were observed between WT, *p53-*null, and *p53^ΔEC^* mice in room air, only the room air WT mice were included in this analysis. For the *p53^ΔEC^* samples in hyperoxia, we sequenced 2 sets of mice, which we refer to as *p53^ΔEC1^ or p53^ΔEC2^*, each containing a male and a female. Notably, there was an increase in the early Cap2 cell population, indicating more conversion from Cap1 to Cap2 (Figure 7, A and B, and Supplemental Table 1). Strikingly, a new population of ECs emerged in the *p53^ΔEC^* mice in hyperoxia consisting of approximately 15% of all ECs (Figure 7, A and B). This distinct cell cluster — which we refer to as “transitional EC” — emerged from Cap1 cells and occupies an intermediate transcriptional position between early and mature Cap2 cells, as predicted by Monocle trajectory analysis (36–40) (Figure 7C and Supplemental Figure 8E). Differential gene analysis between hyperoxia control and *p53^ΔEC^* mice revealed upregulation of some growth factors such as placental growth factor (*Pgf*) and growth differentiation factor 15 (*Gdf15*), both of which have been implicated in BPD (41–43) (Figure 7D and Supplemental Table 4). It also showed upregulation of angiogenesis-related genes, such as angiopoietin 2 (*Angpt2*), and genes associated with oxidative stress, like oxidative stress–induced growth inhibitor 1 (*Osgin1*) (44, 45) (Figure 7D). Gene ontology analysis of the upregulated genes in transitional ECs confirmed that these cells might be regulating oxidative and metabolic stress while regulating other cell populations like fibroblasts (Supplemental Figure 8A). Because some of these genes have been associated with senescence, such as *Gdf15*, we ran the senMayo and SASP gene score using our scRNA-Seq data (Figure 7E). Both scores seemed to be higher in the *p53^ΔEC^* mice, suggesting a potential phenotype for these transitional ECs. Remarkably, despite expressing endothelial lineage markers such as cadherin-5 (*Cdh5*) and platelet EC adhesion molecule (*Pecam1*), these ECs do not express PLVAP or CAR4, marker genes for Cap1 and Cap2, respectively (Supplemental Figure 8B). The early Cap2 cell cluster, which was expanded in the *p53^ΔEC^* mice, did express PLVAP, potentially contributing to the improvement in Cap1 vasculature we observed (Supplemental Figure 8B). Additionally, most proliferating cells are Cap1 cells and express PLVAP (Supplemental Figure 8B).

To validate the presence of this transitional EC state, we performed RNAscope for *Gdf15*, a specific marker present in the transitional ECs (Supplemental Figure 8F), in combination with immunofluorescence for ERG, an EC nuclear marker. Thus, we were able to show upregulation of *Gdf15* in ECs in the *p53^ΔEC^* mutant group compared with the control hyperoxia group (Figure 7F), consistent with the emergence of a transitional EC. Relevant to these findings, loss of *Gdf15* in neonatal hyperoxia has been shown to result in increased mortality and worsen alveolarization and vascular development, highlighting the importance of transitional EC genes in injury response (42).

We conducted further analysis for *Cdkn1a* (*p21*) RNA levels in the scRNA-Seq data to verify gene deletion and explore the mechanisms for the appearance of this new cluster. Comparison of *p21* across samples revealed that although Cap1 cells in the hyperoxia *p53^ΔEC^* group indeed showed similar levels to the Cap1 cells in room air, Cap2 cells and transitional cells had high levels of *p21*, comparable to the control mice in hyperoxia (Supplemental Figure 8C). Additionally, KEGG pathway analysis indicated an upregulation in senescence-related genes (Supplemental Figure 8D). A possible explanation is that the few escapers we observed (Figure 6A and Supplemental Figure 7D) have high expression of *p21* or that *p21* itself is being activated by an independent signaling mechanism in the *p53^ΔEC^* in hyperoxia. In fact, *p21* has been shown to be regulated by multiple factors outside of *p53*, including transforming growth factor β (*Tgfb*) (46), growth factors (47), and activin A (*Inhba*) (48).

Given that we did not observe changes in the epithelial deletion of *p53*, we considered potential mesenchymal and immune cell contributions, which also upregulate *p53* in hyperoxia (Supplemental Figure 7F). To narrow down candidates, we used CellChat in scRNA-Seq data to infer cell-cell communication through ligand-receptor interactions (49). Using only Cap1, Cap2 and transitional ECs as targets, we identified interactions arising from mesenchymal and immune cells (Supplemental Figure 9A and Supplemental Table 5). We then compared ligand-receptor interactions between the *p53^ΔEC^* and the *p53-*null conditions to detect signals that could explain their conflicting phenotypes (Supplemental Figure 9, A–D, and Supplemental Table 5). Indeed, we found unique and upregulated interactions in both mouse models upon hyperoxia, with stronger communication between mesenchymal cells and ECs and alveolar macrophages on the immune lineage. Interestingly, most of the ligand-receptor interactions were shared for these 3 EC types; some of these, like adrenomedullin *—* calcitonin receptor-like receptor (*Calcrl* , activated by ligand *Adm*) — have been associated with EC survival, angiogenesis, and inhibition of apoptosis (50). Others, like *Pgf — Vegfr1,* insulin-like growth factor 2 (*Igf2*), and IGF-1-receptor (*Igf1r*), *Angpt2*, and *Tek —* have been associated with the regulation of angiogenesis (51, 52). TGF-β, an important pathway in lung development that has been implicated in the pathogenesis of BPD and a *p53*-independent activator of *p21* (46, 53), was one of the shared interactions detected. Overall, this analysis indicates that non-EC contributions could influence vascular survival and repair upon hyperoxia. Furthermore, it suggests that several pro- and antiangiogenic signals could be modulating Cap1-to-Cap2 cell conversion in the *p53^ΔEC^* mice, resulting in more Cap1 cells and improved vasculature.

**Figure 8:**
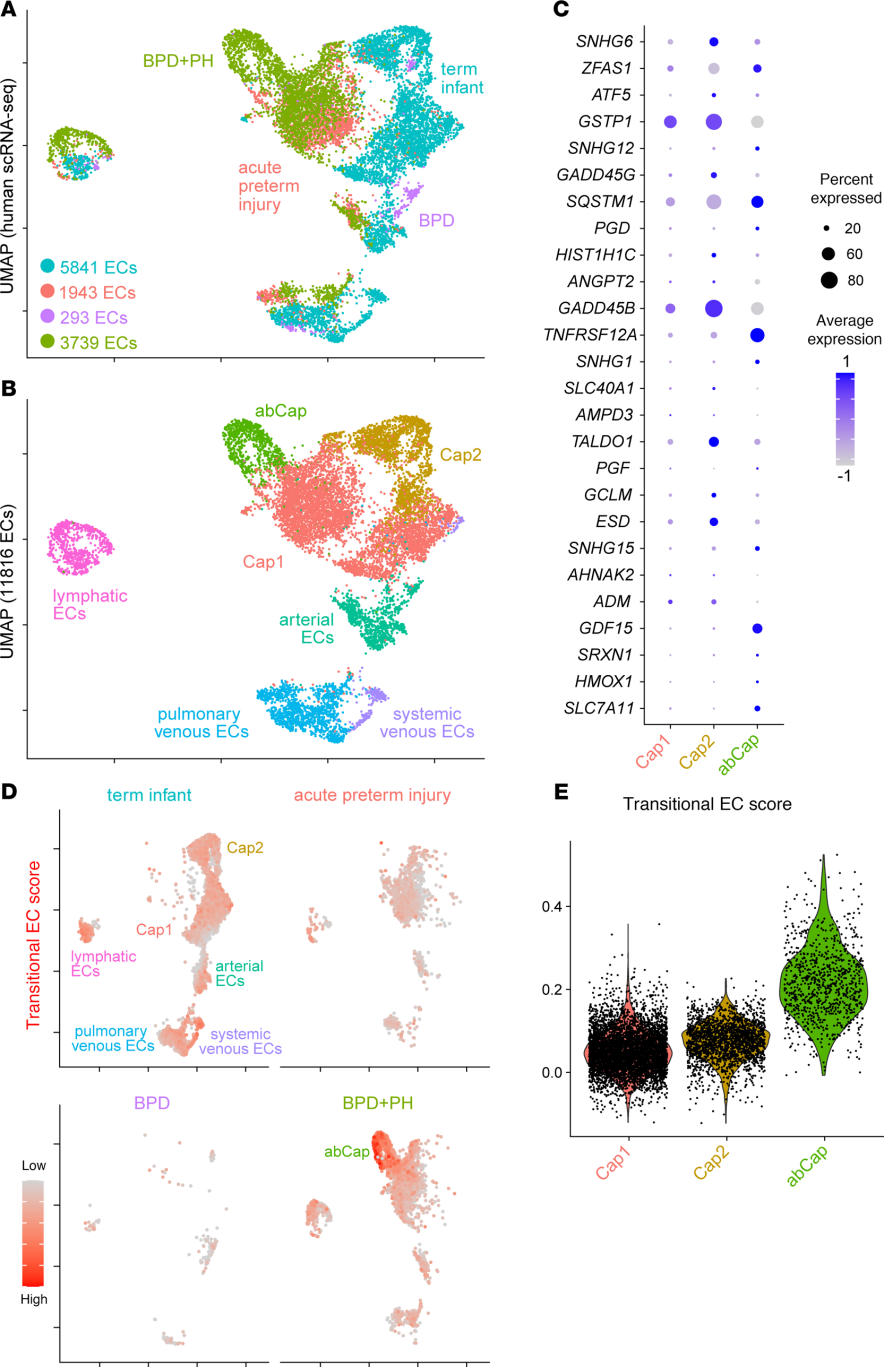
scRNA-Seq analysis of neonatal human lungs with BPD+PH reveals an abCap state that shares a transcriptional signature with transitional ECs identified in the hyperoxia mouse model. (**A**) UMAP of neonatal human lung ECs color coded according to disease status. (**B**) UMAP of pooled neonatal human lung ECs color coded according to cell population. The subcluster representing an abCap state is only present in BPD+PH. (**C**) Dot plot of the top 26 genes upregulated in mouse transitional ECs showing their expression in Cap1, Cap2, and abCap cells. Several of these genes are enriched in abCaps. (**D**) Gene score based on differentially expressed genes found in transitional ECs. This gene signature is upregulated in the abCap cluster in BPD+PH lung ECs compared with ECs in term infants. (**E**) Transitional EC score for Cap1, Cap2, and abCaps generated from scRNA-Seq data demonstrating that abCaps have a gene signature that resembles transitional ECs more closely than Cap1 or Cap2 cells. abCap, aberrant capillary endothelial cells; BPD, bronchopulmonary dysplasia; PH, pulmonary hypertension.

Recently, scRNA-Seq data from neonatal human lungs of BPD and its most severe form, which is accompanied by pulmonary hypertension (BPD+PH), have become available. Notably, within the BPD+PH samples, ECs exhibit an aberrant capillary cell state (abCap) characterized by a loss of hallmark marker genes that define both Cap1 and Cap2 cells (54). To explore the potential relevance of our findings to human disease, we reanalyzed this dataset and performed subclustering of the EC populations (Figure 8, A and B). We next examined the expression patterns of the top 26 genes enriched in our identified transitional ECs, comparing their levels across Cap1, Cap2, and abCap populations (Figure 8C). Strikingly, several of these genes were predominantly expressed within the abCap cluster. To further assess this relationship, we generated a gene score based on the differentially expressed genes from the transitional EC population in our model. Surprisingly, this transitional EC gene signature was elevated in the abCap population of human BPD+PH lungs (Figure 8, D and E), underscoring the biological and clinical relevance of our findings and suggesting conserved mechanisms of endothelial plasticity across species.

## Discussion

In this study, we demonstrated that neonatal hyperoxia targets Cap2 cells, leading to their demise and subsequent replacement by Cap1 cells, resulting in Cap1 cell depletion and contributing to the vascular rarefaction seen in a mouse model of BPD. Our findings also highlight the pivotal role of the p53 pathway in orchestrating EC regeneration and Cap2 specification during hyperoxic injury-repair. Through global deletion of *p53*, we observed worsened vascular phenotypes, implicating *p53* in mediating vascular recovery. Conversely, EC-specific deletion of *p53* reversed the vascular phenotype and improved alveolar simplification. This recovery was associated with the emergence of a transitional EC state between Cap1 and Cap2 fate, characterized by the upregulation of growth factors and oxidative stress response genes. These findings indicate that *p53* is required to maintain lineage fidelity during the Cap1-to-Cap2 transition under hyperoxic conditions; in its absence, a new transitional EC state emerges. Importantly, we also show that these transitional ECs have a similar transcriptional profile to an aberrant cell state that is present in humans with BPD+PH, suggesting that mechanisms for endothelial repair are conserved among species. Our study provides insights into the cellular dynamics underlying vascular injury and repair in BPD, shedding light on the role of EC heterogeneity and the p53 pathway in this context.

Our work contributes to the growing body of literature elucidating the molecular mechanisms underlying BPD pathogenesis. By delineating the cellular dynamics of EC subpopulations and the p53 pathway in vascular injury and repair, we believe we provide novel insights that could inform the development of targeted therapeutic strategies for BPD. Although it has not been tested, it is possible that ablating the p53 pathway in the endothelium could promote vascular resiliency and recovery for both the vasculature and alveolar epithelium in cases of severe BPD.

Moreover, the identification of the transitional EC state expands our understanding of the cellular heterogeneity within the pulmonary vasculature, drawing remarkable comparisons to lung epithelial regeneration and repair. Analogous to the lung epithelium, where damage-associated transient progenitors (DATPs) have been characterized, our study highlights the functional significance of Cap1 cells as endothelial stem-like cells in injury contexts, akin to the role of AT2 cells in epithelial repair (26–28). This parallelism extends to the regulation of cell state by the p53 pathway; DATPs have been described to upregulate p53 target genes (26–28), and, more recently, the role of *p53* in promoting AT1 differentiation has been demonstrated (55), thereby highlighting intriguing similarities in the regulatory mechanisms governing cellular plasticity across different pulmonary cell lineages.

While our study provides compelling evidence for the involvement of the p53 pathway in the vascular phenotype of BPD, further studies are warranted to investigate the precise mechanisms by which *p53* regulates EC regeneration and Cap2 specification, including non-cell autonomous mechanisms. Our data also underline the interplay between different cell types and how angiocrine signaling could play a role in injury-repair. The functional significance of the transitional EC state identified in our study requires further investigation to determine its role in vascular repair. Moreover, the conversion of Cap1 to Cap2 fate necessitates a closer look to further dissect the balance between pro- and antiangiogenic signals that achieve appropriate vascular regeneration.

Additionally, further experiments are needed to better understand the injury mechanism of oxygen and activation of *p53* in this context. Historically, reactive oxygen species (ROS) have been identified as the main culprits for DNA damage in hyperoxia, which, in turn, upregulate *p53* (21, 22, 56). However, changing superoxide levels to decrease oxidative stress has not been sufficient to rescue oxygen toxicity (57). More recent studies emphasize the effects of oxygen in mitochondrial and cellular metabolism and attribute the damage to degradation of the electron transport chain (ETC) (58). Mitochondrial dysfunction can, in turn, activate *p53* to reduce induced stress response (ISR) genes; alternatively, ISR genes can themselves induce *p53* (59). It would be interesting to test whether this mechanism is involved in endothelial hyperoxic injury and if it can be rescued by targeting the different complexes of the ETC.

In conclusion, our findings underscore the importance of EC heterogeneity and *p53* in mediating vascular injury and repair in BPD. By elucidating the cellular dynamics underlying BPD pathogenesis, our study lays the foundation for future research aimed at understanding cell fate choice and regeneration during development and disease.

## Methods

### Sex as a biological variable.

Our study included both male and female mice. No sex-specific differences were observed.

### Antibodies.

Antibodies used are outlined in Supplemental Table 6.

### Mouse strains and hyperoxia treatment.

The following mouse strains were used (Jackson Lab strain): *Trp53^flox/flox^* (24) (#008462) was obtained from Guillermina Lozano (The University of Texas MD Anderson Cancer Center), *CMV-Cre* (23) (#003465), *Tek-Cre* (27) (also called *Tie2-Cre*) (#008863), *Cdh5-CreER* (26) was obtained from Ralf Adams (CancerTools), *Shh^Cre^* (28) (#005622), *Kit^CreER^* (14) was gifted by Dieter Saur (Technical University of Munich), *Car4^CreER^* (17) (#038602), *Rosa^Sun1GFP^* (15) (#021039), and *Rosa^tdT^* (16) (#007914). WT C57BL/6J (#000664) mice were used to establish a control hyperoxia phenotype and in scRNA-Seq. Mice of both sexes were used, and the number of control-mutant pairs are listed in the figure legends. Tamoxifen (T5648; Sigma) dissolved in corn oil (C8267; Sigma) was administered to pups at P0 via intraperitoneal injection to induce Cre-Lox recombination when required; specific doses are noted in the figures. Hyperoxia exposure was performed following published protocols with minor modifications (4). Littermates were either maintained in room air (21% O_2_) or placed in normobaric 80% O_2_ at P0 or P1 if not injected, or P2 if injected, until the date of harvest. A sealed animal chamber (BioSpherix, A30274P) was used to house hyperoxia cages, in which the O_2_ concentration was maintained using an oxygen controller (BioSpherix, P360) connected to an oxygen source. To prevent excessive oxidative stress in the hyperoxia condition, nursing dams were switched between room air and hyperoxia conditions every 24 hours for the duration of exposure.

### Section immunostaining.

Lungs were harvested as described in our previous publications (6). Mice were first anesthetized with Avertin (T48402, Sigma), then perfused through the right ventricle with PBS. The trachea was then cannulated, and the lung was inflated using 0.5% paraformaldehyde (PFA, Thermo Scientific, J19943.K2) in PBS at 25 cm H_2_O pressure. After extraction, the lung was fixed in 0.5% PFA at room temperature for 3–6 hours, then briefly washed and left in PBS overnight at 4°C. Section immunostaining was performed as described in our previous publications (6). Fixed lobes were cryoprotected in 20% sucrose in PBS with 10% OCT (Tissue-Tek, 4583) and frozen in OCT blocks. Sections of 20 μm thickness were blocked in PBS + 0.3% Triton X-100 and 5% normal donkey serum (Jackson ImmunoResearch, 017-000-121) for 1 hour. Primary antibodies in PBS + 0.3% Triton X-100 were added to the sections and incubated at 4°C overnight in a humidified container. Sections were washed for 30 minutes in a Coplin jar with PBS, then incubated with donkey secondary antibodies and 4’,6-diamidino-2-phenylindole (DAPI) diluted in PBS + Triton X-100 at room temperature for 1 hour. Following another PBS wash in a Coplin jar, sections were mounted with Aqua-Poly/Mount (Polysciences, 18606). Images were taken with either an Olympus FV1000 confocal microscope using a 30X oil objective or a Nikon Ti2 with AX Confocal System microscope using a 20X air objective or 60X oil objective at a resolution of 1,024, 2,048, or 4,096 (Supplemental Figure 10, A and B).

### Whole-mount immunostaining.

Whole-mount immunostaining was performed as described in our previous publications (6). Thin strips 3 mm in length were cut from the distal edge of the cranial lobe, then blocked in PBS + 0.3% Triton X-100 and 5% normal donkey serum for 1 hour at room temperature. Strips were added to a solution of primary antibodies diluted in PBS + 0.3% Triton X-100 and rocked overnight at 4°C. The strips were then washed 3 times in PBS + 0.3% Triton X-100 + 1% Tween-20 (PBSTT) at room temperature for 1 hour each wash. Secondary antibodies and DAPI were diluted in PBS + 0.3% Triton X-100, and strips were placed in the solution and rocked overnight at 4°C. On the third day, the strips were washed 3 times in PBSTT as before, then post-fixed in 2% PFA in PBS for 2 hours. After fixation, the strips were mounted flat side up with Aqua-Poly/Mount. Images were taken with either an Olympus FV1000 confocal microscope or a Nikon Ti2 with AX Confocal System microscope using a 20X air objective or 60X oil objective at a resolution of 1,024 or 2,048. Z-stack images with a step size of 0.5 or 1 μm were acquired beginning at the top of the tissue with a depth of 20 μm (Supplemental Figure 10, A and B).

### Vasculature analysis and quantification.

To analyze and quantify CAR4, PLVAP, and ICAM2 vasculature staining, whole-mount immunostained strips were used to ensure comparable, accurate rendering of the vessel surfaces between samples. An Olympus FV1000 confocal microscope was used to image samples with a 30X oil objective and a field size of 424 μm × 424 μm × 20 μm and a pixel dimension of 1,024 x 1,024 x 20 (Supplemental Figure 10, A and B). Three Z-stacks per strip were analyzed with Imaris software to render a surface to measure vascular surface area. To quantity EC number and proliferating cell number, immunostained sections were imaged on the same objective with a field size of 424 μm × 424 μm × 1 μm and a pixel dimension of 1,024 × 1,024 (Supplemental Figure 10, A and B). Imaris surface rendering software was again used to quantify EC number (ERG) and proliferating cell number (KI67) from at least 3 images per section (Supplemental Figure 10C). Following quantification, GraphPad Prism 10 was used to generate plots and for statistical analysis.

### MLI analysis.

MLI was measured on 5 μm thick frozen lung sections stained with H&E. This quantification was performed in Photoshop in accordance with a published protocol (60). For each mouse lung, 3 images were acquired on an upright Olympus BX60 microscope with a 10X objective. The guide function of Photoshop was used to add 2 evenly spaced vertical and horizontal grid lines on each picture. Along the grid lines, the distance between one alveolar wall and the other was measured using the Photoshop ruler tool. Airways, large vessels, and alveoli in which one wall was not visible were excluded. At least 40 intercepts per image and 3 images per mouse were measured. GraphPad Prism 10 was used to plot MLI values and for subsequent statistical analysis.

### RNAscope in situ hybridization.

Harvested lungs were fixed and washed as above and immediately placed in 20% sucrose with 10% OCT (Tissue-Tek, 4583) and 0.5% PFA in PBS overnight at 4°C, then frozen into OCT blocks. Sections of 10 μm thickness were probed for *Cdkn1a* and *Gdf15* (Advanced Cell Diagnostics) using the Multiplex Fluorescent Detection Kit v2 (Advanced Cell Diagnostics, 323110). Sections were treated with 4% PFA for 10 minutes, washed with DEPC-H_2_O and 100% ethanol, then treated with hydrogen peroxide for 10 minutes and washed with DEPC-H_2_O. Slides were submerged in boiling 1X target retrieval solution for 4 minutes and treated with Protease III for 30 minutes at room temperature. Probes were added for 2 hours at 40°C and the slides were left in 5X SSC buffer (Thermo Scientific, J60839.K3) overnight. Following a quick wash in RNAscope wash buffer, slides were treated with Amp1 for 30 minutes, Amp2 for 30 minutes, and Amp3 for 15 minutes at 40°C for each treatment with intermittent washes. HRP-C1 was added at 40°C for 15 minutes, then OPAL 520 (1:1500 in TSA buffer, Akoya Biosciences, FP1487001KT) at 40°C for 30 minutes, and finally HRP blocker for 15 minutes at 40°C with intermittent washes. This same process was repeated for HRP-C2 and OPAL 570 (Akoya Biosciences, FP1488001KT),and for HRP-C3, OPAL 690 (Akoya Biosciences, FP1497001KT). Following RNAscope, sections were immunostained as described above if required. Images were acquired on either an Olympus FV1000 confocal microscope using a 60X oil objective or a Yokogawa CSU-W1 confocal microscope on a Nikon Ti2 Eclipse microscope stand also using a 60X oil objective at a resolution of 2,048.

### Cell dissociation and FACS.

Lung dissociation was performed as described in our previous publications with minor modifications (6). Whole lungs were harvested in Leibovitz’s Medium (Gibco, 21-083-027), minced with forceps, and digested in Leibovitz’s with 2 mg/mL Collagenase Type I (Worthington, CLS-1, LS004197), 2 mg/mL Elastase (Worthington, ESL, LS002294), and 0.5 mg/mL DNase I (Worthington, D, LS002007) for 30 minutes at 37°C. After 15 minutes of digestion, the tissue was mechanically agitated by pipetting. Fetal bovine serum (FBS, Invitrogen, 10082-139) was then added to a final concentration of 20%, and the solution was homogenized. Samples were immediately transferred to the 4°C cold room on ice, where they were filtered with a 70 μm cell strainer (Falcon, 352350). Strained samples were centrifuged at 1,677 g for 1 minute and resuspended in 1 mL red blood cell lysis buffer (15 mM NH4Cl, 12 mM NaHCO3, 0.1 mM EDTA, pH 8.0) for 3 minutes. Samples were again centrifuged at 1,677 g for 1 minute, washed with Leibovitz’s + 10% FBS, and filtered into a 5 mL tube with cell strainer cap (Falcon, 352235). Cells were then incubated with CD45-PE/Cy7 (BioLegend, 103114), ICAM2-A647 (Invitrogen, A15452), and ECAD-A488 (eBioscience, 53-3249-82) at a concentration of 1:250 for 30 minutes, then centrifuged as above and washed with Leibovitz’s + 10% FBS. Samples were refiltered and incubated with SYTOX Blue (Invitrogen, S34857), then sorted on a BD FACSAria II Cell Sorter. Excluding dead cells and doublets, 4 cell populations were collected: CD45^+^ as the immune lineage, ICAM2^+^ (from CD45^–^) as the endothelial lineage, ECAD^+^ (from CD45^–^ and ICAM2^–^) as the epithelial lineage, and triple negative representing the mesenchymal lineage.

### scRNA-Seq.

Sequencing and analysis were performed as described and raw data was deposited in GEO (6) (GSE266988). FACS-purified cells from each lineage were combined in equal proportions into a single tube and prepared for sequencing using the Chromium Single Cell Gene Expression Solution Platform (10x Genomics). Chromium scRNA-Seq output was processed with Cell Ranger, and subsequent analysis was performed using R packages Seurat (61) (v5.0.1), Monocle3 (v1.2.9), and CellChat (v1.6.1). Cells were filtered by their gene count to exclude those with counts lower than 200 and higher than 6,000, and more than 10% of mitochondrial genes; both parameters were adjusted depending on each sample. We used FindIntegrationAnchors and IntegrateData to aggregate multiple data sets. Cell lineages were established based on the expression of *Cdh1* (epithelium), *Cdh5* (endothelium), *Col3a1* (mesenchyme), and *Ptprc* (immune). Doublets were identified based on the coexpression of these markers and then eliminated. ECs were subsetted and reclustered and Findmarkers was used to do differential gene analysis between cluster or subpopulations. Enhanced Volcano 1.14.0 was used for plotting with a fold change cutoff of 1 and a *P* value cutoff at 10e-5. Monocle3 and SeuratWrappers were used to analyze capillary ECs and generate pseudotime trajectories. Finally, CellChat objects were created per sample and then merged to perform differential gene expression analysis and extract ligand receptor pairs that were upregulated. For time point comparisons (Supplemental Figure 7B), Seurat was used on the published datasets GSE124325 (6). Transitional EC gene score was created by taking the differentially expressed genes with an adjusted *P* value of < 0.05, log fold change of >1.5 and percent expressed of at least 0.5.

### Western blotting.

Mice were treated with 80% O_2_ or room air for 3 days beginning at P1. Lungs were dissected at P4 and lobes were homogenized using a Dounce homogenizer containing 500 μL lysis buffer (100 mM NaCl, 20 mM Tris-HCl, 0.5% NP-40, 1 mM EDTA, 0.5% Triton X-100, 1 mM PMSF, 1 mM NaF, 5 mM NaVO3, 0.1% Halt protease and phosphatase inhibitor cocktail; Thermo Fisher Scientific, 78441). Samples were kept on ice for 10 minutes and then centrifuged at 13,148 g for 10 minutes at 4°C. The supernatant was collected and the protein concentration was determined using the Pierce BCA Protein Assay Kit (Thermo Fisher Scientific, A55865). Sample lysates were reduced with Invitrogen 4X Bolt LDS Sample Buffer (Thermo Fisher Scientific, B0008) supplemented with 50 mM Invitrogen 10X Bolt Sample Reducing Agent (Thermo Fisher Scientific, B0004), diluted to a final concentration of 1X, and denatured by heating to 70°C for 8 minutes. 15 μg of protein in 15 μL lysis buffer/Sample Buffer were loaded on Invitrogen Bolt Bis-Tris Plus Mini Protein Gels, 4%–12%, 1.0 mm, WedgeWell format (Thermo Fisher Scientific, NW04122BOX) alongside 5 μL of the Precision Plus Protein Kaleidoscope ladder (Bio-Rad, 1610375). Proteins were separated at a constant 200V for 35 minutes in 1X buffer containing Invitrogen 20X Bolt MOPS SDS Running Buffer (Thermo Fisher Scientific, B0001) and 0.25% Invitrogen Bolt Antioxidant (Thermo Fisher Scientific, BT0005). Proteins were then transferred to PVDF membrane at 20 V for 60 minutes in 1X transfer buffer made with Invitrogen Bolt Transfer Buffer (20X) and 0.1% Invitrogen Bolt Antioxidant (Thermo Fisher Scientific, BT0005). Membranes were blocked in 5% Bovine Serum Albumin (Dot Scientific Inc., DSA30075) in TBST for 1 hour at room temperature with constant agitation. Primary antibodies (listed above) were added and incubated overnight at 4°C with constant agitation. Membranes were washed in tris-buffered saline with 0.1% Tween 20 (TBST) for 10 minutes a total of 3 times. The following secondary antibodies were added at the stated concentrations, and membranes were incubated for 1 hour at room temperature with constant agitation: Peroxidase AffiniPure Donkey Anti-Rabbit IgG (H+L) (1:10,000, Jackson ImmunoResearch, 711-035-152), Peroxidase AffiniPure Donkey Anti-Mouse IgG (H+L) (1:10,000, Jackson ImmunoResearch, 715-035-151). Membranes were washed in TBST for 10 minutes a total of 5 times. Finally, proteins were detected by covering membranes with SuperSignal West Pico PLUS Chemiluminescent Substrate (Thermo Fisher Scientific, PI34577) for 1 minute and imaging on a Bio-Rad ChemiDoc MP imaging system.

### Large language models.

ChatGPT4 (OpenAI) was used (February - June 2024) to review and suggest edits for portions of the text. Prompts included requests to highlight suggested changes, evaluate strengths and weaknesses of the discussion, identify unclear concepts, and propose introduction and discussion outlines based on results. Additionally, ChatGPT was used to troubleshoot coding related to CellChat with Seurat 5, specifically to clarify library requirements and interpret a single error message.

### Statistics.

For each quantification, the mean was calculated using at least 2, but typically 3–5, representative images from a single lung. A sample size of at least 3 lungs was obtained for each condition. The means were compared across conditions, and statistical testing was performed to determine significance. In comparisons involving only 2 groups (i.e., control room air versus hyperoxia), the Student’s 2-tailed *t* test was used. In comparisons involving control/mutant pairs in room air and hyperoxia, 1-way ANOVA with Tukey’s multiple comparisons was used. Standard deviations for each group are displayed as error bars for each condition; *P* values lower than 0.05 were considered significant.

### Study approval.

All protocols used for this research complied with and were approved by the Institutional Animal Care and Use Committee regulations of MD Anderson Cancer Center and Northwestern University.

### Data availability.

Raw scRNA-Seq data can be found in GEO (GSE266988). The Supporting Data Values file contains all quantifications performed and showed in plots. Supplemental tables include differentially expressed gene lists from each scRNA-Seq shown in volcano plots and CellChat plots.

## Author contributions

LVE, JMSS, and JC designed the research. LVE, JDB, AC, and YL performed the research. LVE, JDB, and AC wrote the paper. All authors read and approved the paper.

## Supplementary Material

Supplemental data

Unedited blot and gel images

Supplemental table 1

Supplemental table 2

Supplemental table 3

Supplemental table 4

Supplemental table 5

Supplemental table 6

Supporting data values

## Version 1 08/05/2025

In-Press Preview

## Version 2 09/09/2025

Electronic publication
