# Supplementary material for: p53 maintains lineage fidelity during lung capillary injury-repair in neonatal hyperoxia
Source: JCI Insight. 2025 Aug 5;10(17):e182880. doi: 10.1172/jci.insight.182880 (PMC12487676; doi:10.1172/jci.insight.182880)
Supplement: Supplemental data [file jciinsight-10-182880-s206.pdf]

SUPPLEMENTAL FIGURES

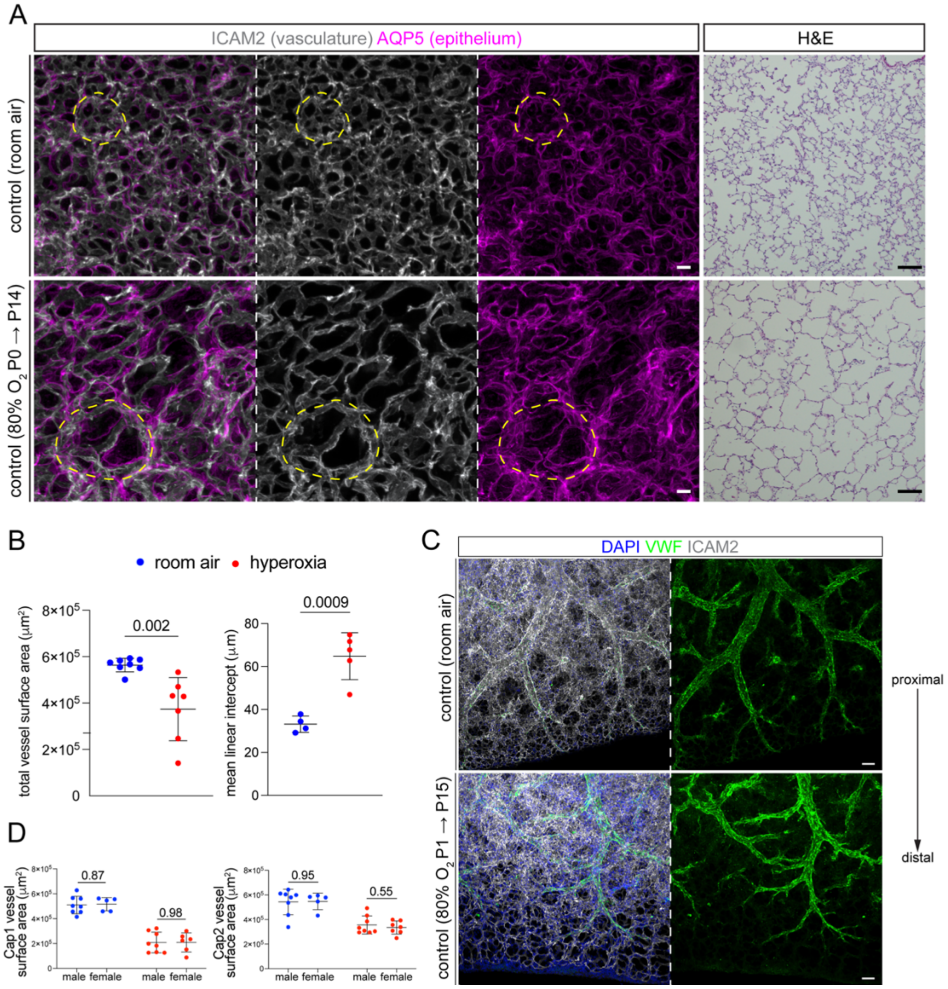

Supplemental Figure 1

(**A**) En-face view of immunostained lungs and H&E sections from room air and hyperoxia conditions. Hyperoxia-treated lungs show alveolar simplification, demonstrated by enlarged alveolar islands (dash; AQP5), and vascular rarefaction, as measured by ICAM2 staining. H&E staining confirms significant enlargement of the airspace. (**B**) Total vessel surface area is quantified based on ICAM2 staining. Enlargement of airspace is quantified by mean linear intercept (MLI) based on H&E staining (Student's t test). (**C**) En-face view of immunostained lungs from room air and hyperoxia conditions showing unaffected morphology of the macrovasculature demonstrated by conserved VWF staining. (**D**) Quantification of Cap1 vessel surface area and Cap2 vessel surface area in room air and hyperoxia after 14 days of exposure divided into male and female mice. No significant sex-dependent differences in vessel area were observed in either condition (Student's t test). For quantification, each symbol represents the average of 3 distinct regions imaged within 1 mouse lung. Images are representative of at least 3 littermate pairs. P, postnatal day. Scale bars, 10 µm (white bars), 100 µm (black bars) (**A**), 50 µm (**C**).

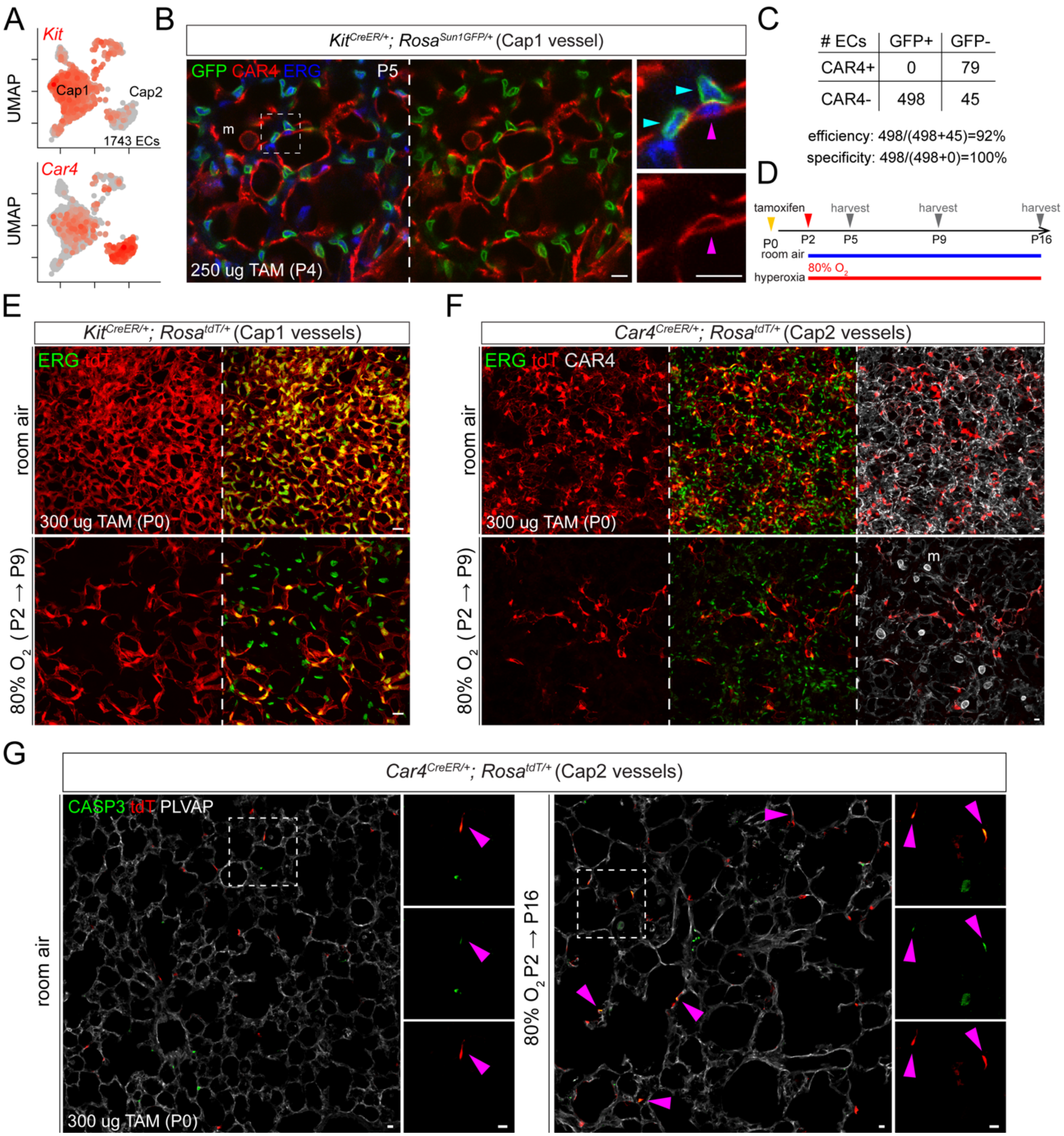

Supplemental Figure 2

(**A**) UMAP of purified lung ECs showing expression of *Kit* and *Car4* as markers for Cap1 and Cap2 ECs, respectively. (**B**) Immunostained lung sections showing the efficiency and specificity of the *Kit*^{CreER} driver. Cap1 ECs are labeled with GFP (cyan arrowheads), while Cap2 ECs (CAR4 positive) lack GFP expression (magenta arrowheads). (**C**) Quantification of the efficiency and specificity of the *Kit*^{CreER} driver. Approximately 92% of all Cap1 ECs were labeled using the

driver, while 100% of GFP labeled cells were Cap1 ECs. (**D**) Experimental model showing tamoxifen administration at P0 in EC type-specific drivers to label Cap1 ECs (*Kit*^{CreER}) or Cap2 ECs (*Car4*^{CreER}) with fluorescent protein tdTomato (tdT) for lineage tracing, followed by 14 days of hyperoxia exposure beginning at P2. Created with BioRender.com. (**E-F**) En-face views of immunostained lungs showing a reduction in Cap1 vessels lineage-traced with the *Kit*^{CreER} driver (**E**) and Cap2 vessels lineage-traced with the *Car4*^{CreER} driver (**F**) after 7 days of hyperoxia exposure. All vessels experience a decrease in cell number, but there is a preferential loss of Cap2 cells as shown by the dramatic reduction in expression of CAR4 and tdT. (**G**) Section view of immunostained lungs showing cleaved-CASP3 in Cap2 vessels. CASP3 frequently co-localizes with the tdT reporter (magenta arrowheads) under hyperoxia conditions, but rarely in room air, confirming that apoptotic cells are of Cap2 lineage. Images are representative of at least 3 littermate pairs. m, macrophage. P, postnatal day. TAM, 250 µg of tamoxifen administered at P4 (**B**), 300 µg of tamoxifen administered at P0 (**E-G**). Scale bars, 10 µm (**B, F, G**), 50 µm (**E**).

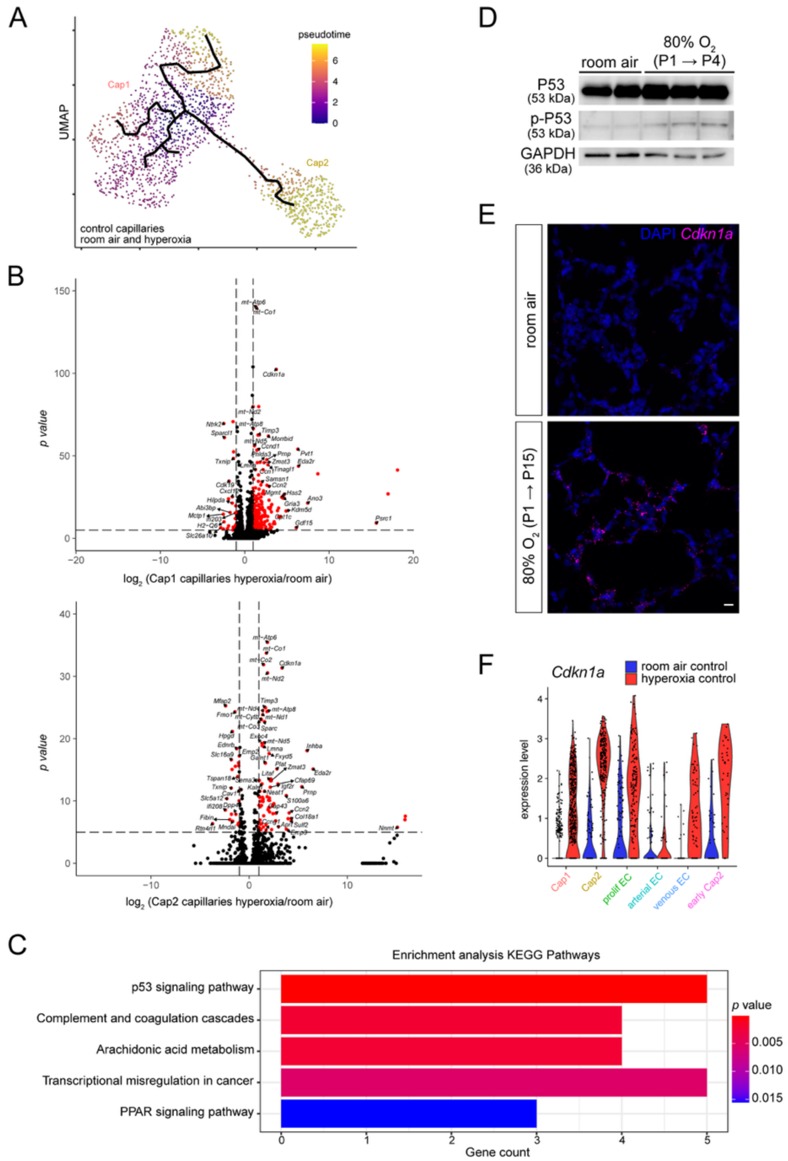

Supplemental Figure 3

(**A**) Pseudotime plot showing the trajectory (black line) of capillary ECs in room air and hyperoxia combined. Dark blue/purple represent pseudotime 0 that co-localize with the initial Cap1 cells; yellow represents fully differentiated Cap2 cells and represent a later pseudotime. (**B**) Volcano Plots showing that upregulation of p53 target genes is conserved in capillary subtypes under hyperoxia conditions. (**C**) Enrichment analysis of the top upregulated genes in capillary ECs under room air and hyperoxia conditions identifying the p53 signaling pathway as highly enriched. (**D**) Western blot depicting increased expression and phosphorylation of P53 after 3 days of hyperoxia exposure compared to room air. GAPDH was used as a loading control. (**E**) RNAscope in situ hybridization of lung sections showing the widespread upregulation of *Cdkn1a* in hyperoxia compared to room air. (**F**) Violin plot showing expression of *Cdkn1a* in each EC population in room air and hyperoxia. *Cdkn1a* is upregulated in every EC population except arteries. Images are representative of at least 3 littermate pairs. P, postnatal day. Scale bars, 10 µm.

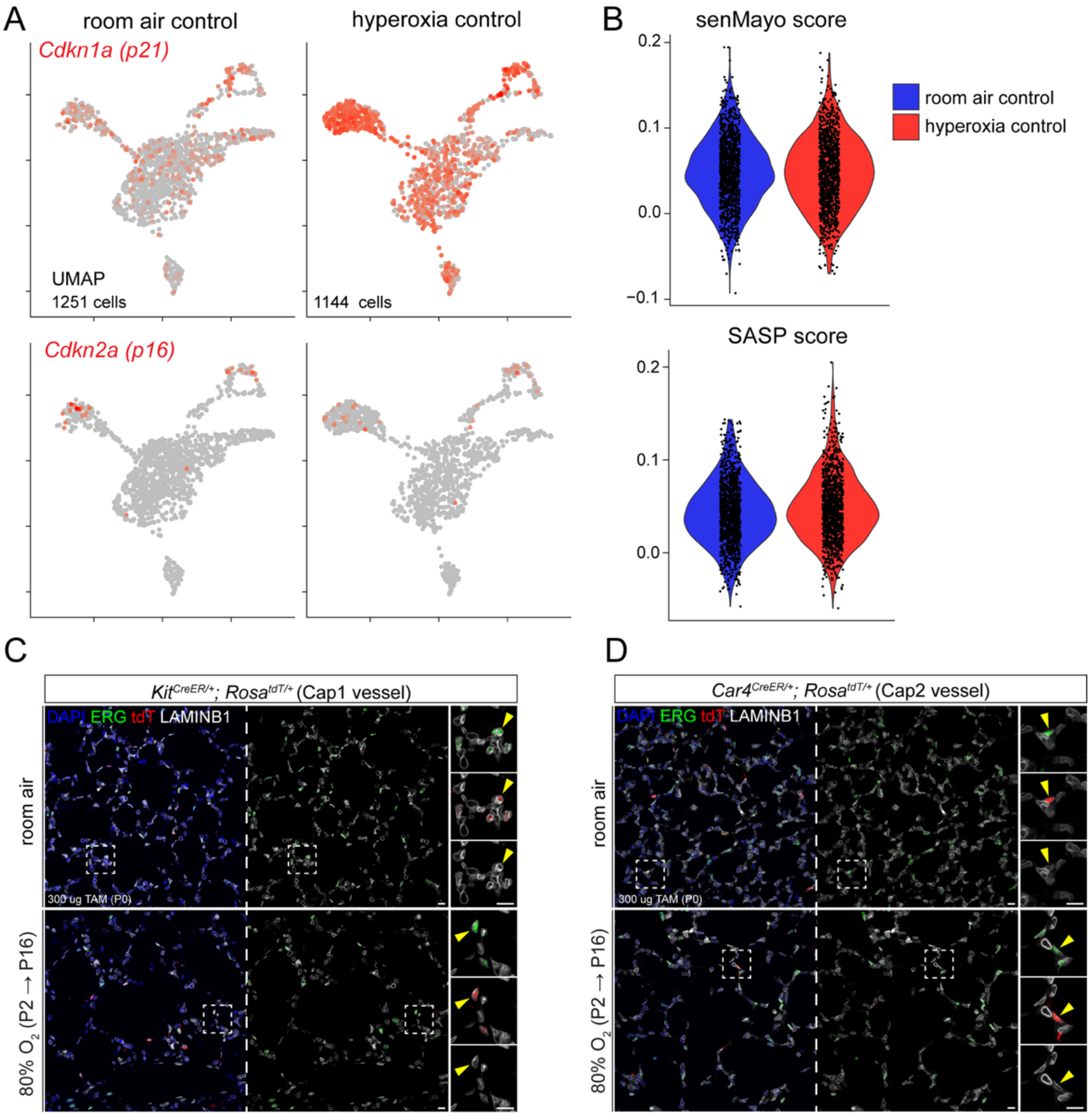

Supplemental Figure 4

(**A**) UMAP of purified ECs showing expression of p53 target genes *Cdkn1a* (p21) and *Cdkn2a* (p16) in room air and 14 days of hyperoxia demonstrating the upregulation of *Cdkn1a* in lung ECs in hyperoxia. Upregulation of *Cdkn2a* is not observed in hyperoxia. (**B**) senMayo gene senescence scores and SASP gene scores are comparable between room air and hyperoxia. ECs in hyperoxia do not show significant upregulation of genes associated with senescence and do not release more SASP factors than ECs in room air. (**C-D**) Section view of immunostained lungs showing that ECs from neither Cap1 (**C**) nor Cap2 (**D**) vessels experience loss of LAMINB1 upon exposure to 14 days of hyperoxia (yellow arrowheads). This suggests that lineage-traced Cap1 and Cap2 cells are not experiencing senescence. Boxed regions are magnified. Images are representative of at least 3 littermate pairs. P, postnatal day. TAM, 300 µg of tamoxifen administered at P0. Scale bars, 10 µm.

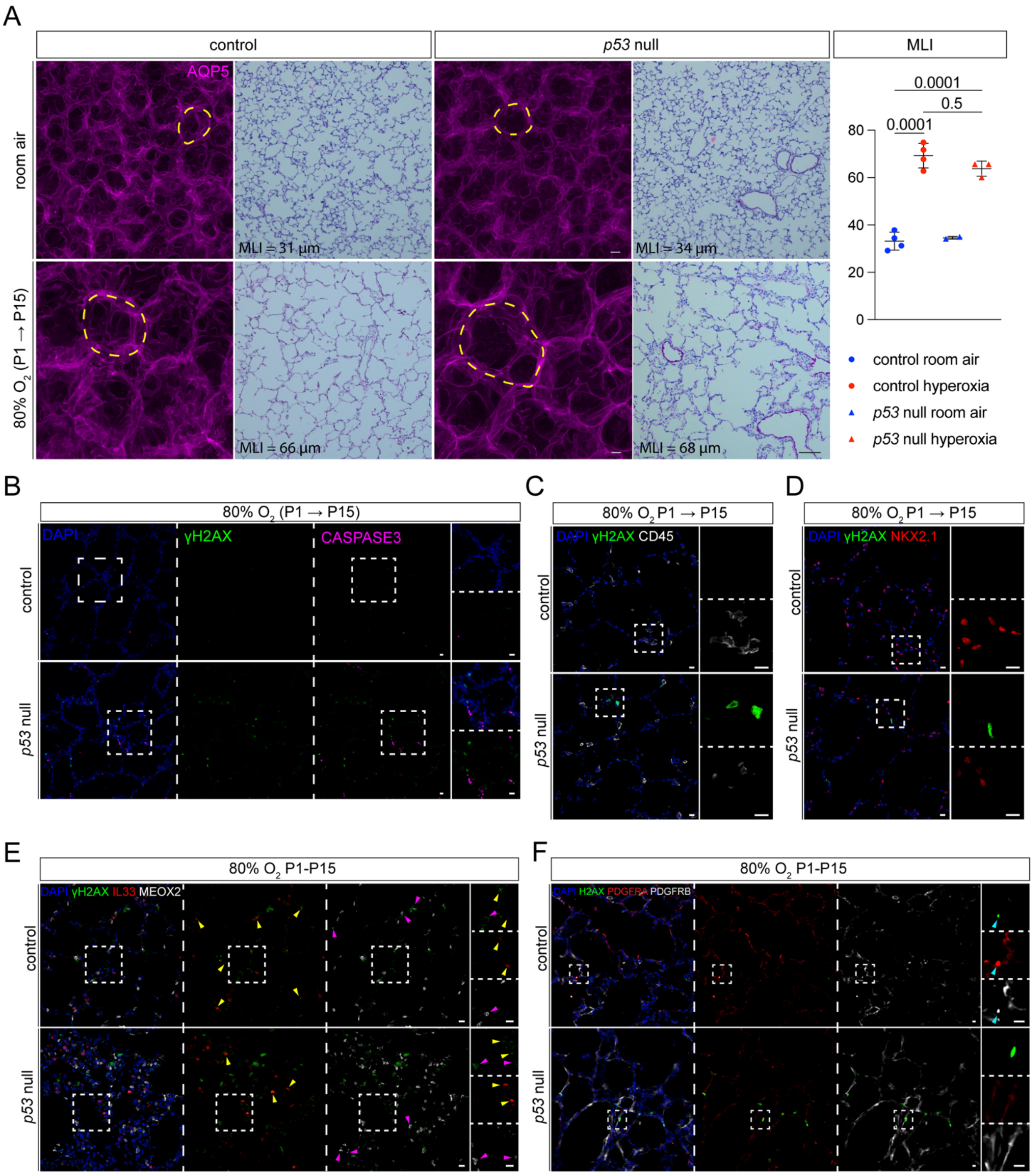

Supplemental Figure 5

(**A**) En-face view of immunostained lungs and H&E-stained lung sections showing the extent of alveolar simplification in each experimental condition, along with associated MLI quantification. AQP5 staining reveals a similar degree of simplification between hyperoxia control and hyperoxia *p53* null, with comparable enlargement of alveolar islands (dashed regions). MLI

quantification confirms airspace enlargement is comparable between hyperoxia control and hyperoxia *p53* null (Student's t test). Each symbol represents the average of 3 distinct regions imaged within 1 mouse lung. (**B**) Immunostained lung sections showing expression of γH2AX and cleaved-CASP3 in hyperoxia control and *p53* null lungs. γH2AX and cleaved-CASP3 are both upregulated in the *p53* null compared to control, but little co-localization was observed between markers. (**C-D**) Immunostained lung sections showing expression of γH2AX and immune cell marker CD45 (**C**) or epithelial cell marker NKX2.1 (**D**) in hyperoxia control and hyperoxia *p53* null lungs. No co-localization of γH2AX with CD45 or NKX2.1 was observed, suggesting DNA damage is not found in immune or epithelial cells upon hyperoxia exposure in the control or *p53* null. (**E-F**) Immunostained lung sections showing expression of γH2AX and mesenchymal markers IL33 and MEOX2 (**E**) or PDGFRα and PDGFRβ (**F**) in hyperoxia control and hyperoxia *p53* null. γH2AX frequently co-localizes with IL33 and MEOX2, but few instances of co-localization with PDGFRα/β were observed, suggesting DNA damage may be specific to certain mesenchymal cells. Boxed regions are magnified. Images are representative of at least 3 littermate pairs. P, postnatal day. Scale bars, 10 μm (white bars), 100 μm (black bars).

A

Gene list for Cap2 cell score

Itgb5	Trim16
Chst1	Tmem47
Sirpa	Tbx3
Tbx2	Agfg1
Car4	Ly6a
Ednrb	Prx
Emp2	Ly6c1
Apln	Timp3
Rtn1	Mgst3
Enho	Stxbp6
Pmp22	Arhgef3
Tppp3	Tspan15
Igfbp7	Rgs12
Cd24a	Rcsd1
Tbxa2r	Bcam
Kitl	Pdlim2
Cyp4b1	Tmcc2
Gem	Mgll
Rasgrp2	Atp13a2
Nhlrc2	Dhrs3
Pvrl3	Nrp1
Kdr	Lhfp
Plaur	Mpped2
Ptp4a3	Pcdh1

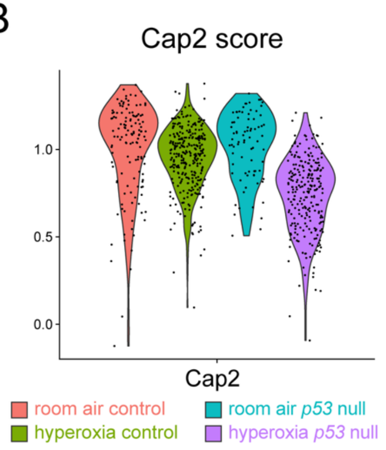

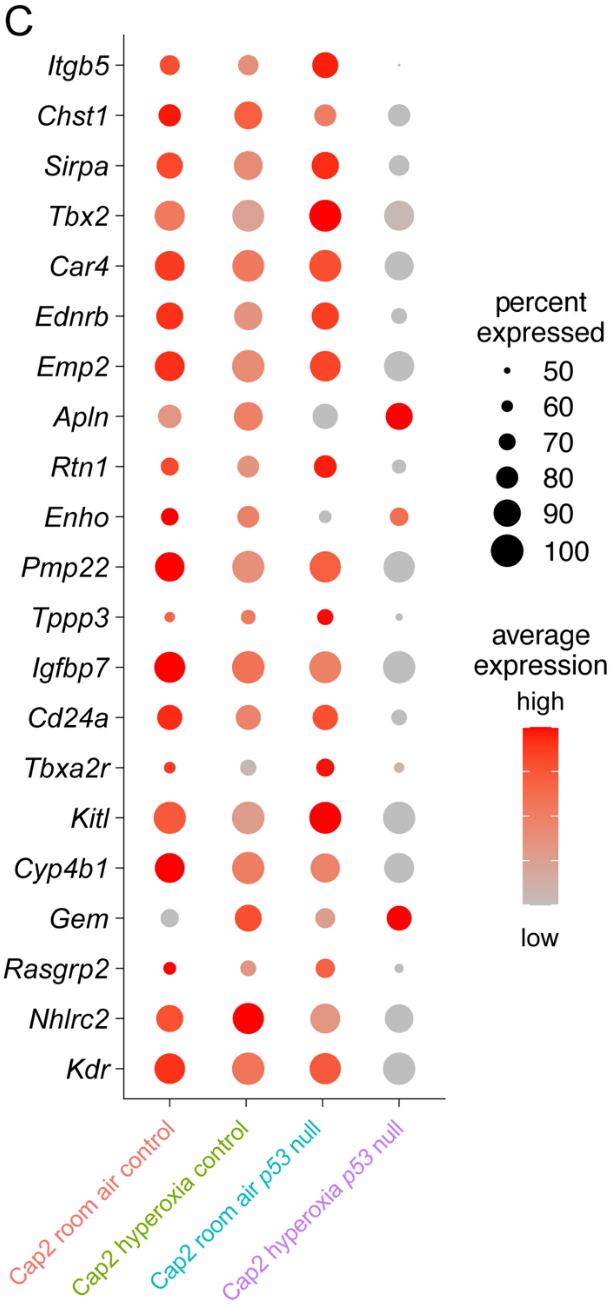

Supplemental Figure 6

(**A**) Gene list of Cap2 EC marker genes expressed at room air at P14 as determined by scRNA-seq that were used to build a Cap2 score. (**B**) Violin plot showing the Cap2 score for each condition which suggests Cap2 cells significantly downregulate their marker genes in the hyperoxia *p53* null compared to all other conditions. (**C**) Dot plot showing the expression of the top 21 Cap2 genes in each condition which are substantially downregulated in the *p53* null lung in hyperoxia.

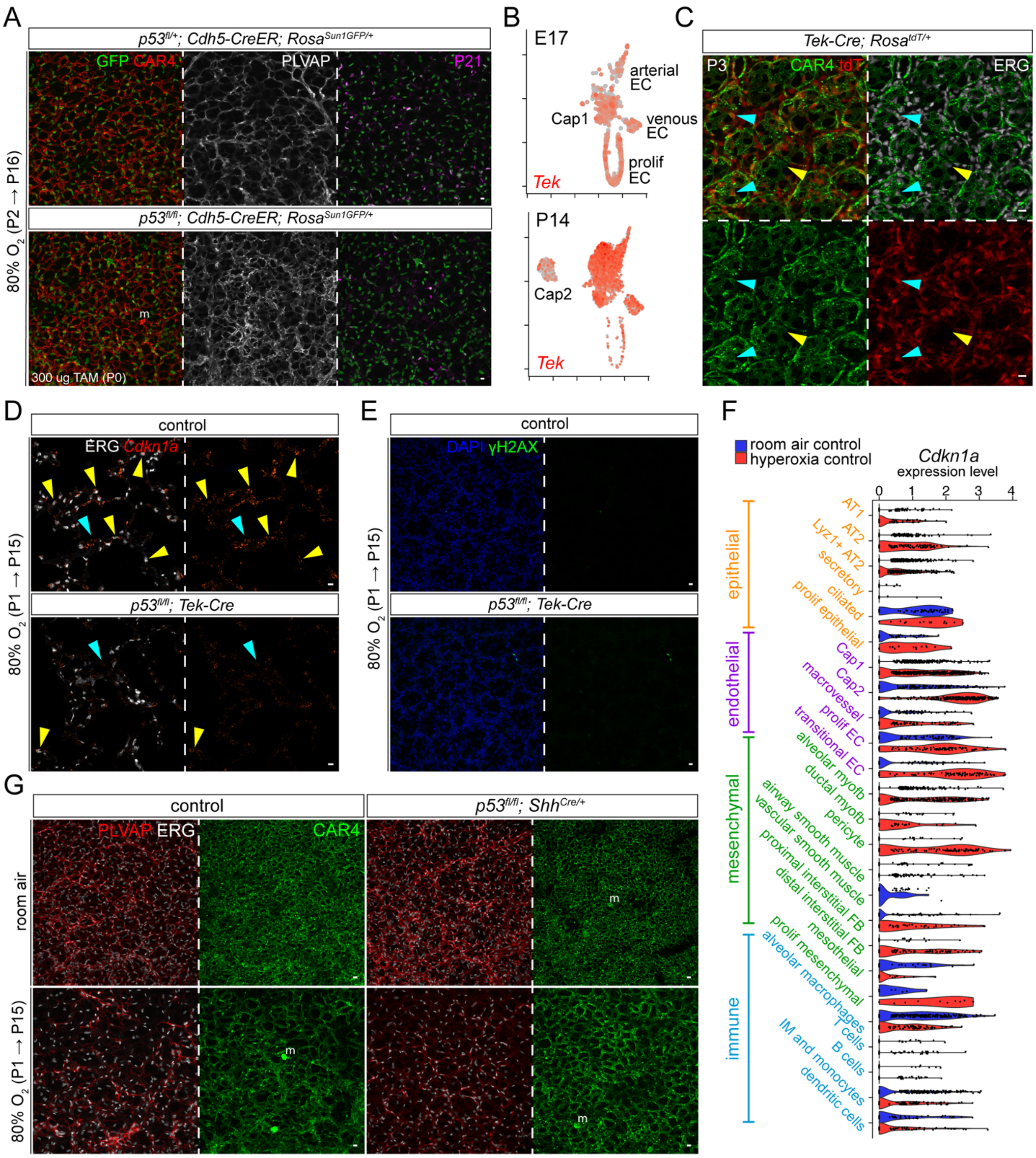

Supplemental Figure 7

(**A**) En-face view of immunostained lungs showing the EC type-specific effect of *p53* deletion using the pan-endothelial *Cdh5-CreER* driver with *Rosa*^{Sun1GFP}. Endothelial deletion of *p53* resulted in an overall improvement in vascular density, most notably in the Cap1 vasculature (PLVAP). This mutant showed incomplete deletion of P21, assessed by GFP+ and P21+ cells. (**B**) UMAP of purified lung ECs showing expression of *Tek* at E17 and P14. Though postnatal Cap2 cells express low levels of *Tek*, it is expressed at E17 in the immature Cap1 population

which gives rise to the Cap2 cells. (**C**) En-face view of immunostained lungs showing expression of tdT in lineage-traced ECs using the *Tek-Cre* driver. ERG revealed the driver to be highly specific to ECs; most Cap2 ECs show expression of tdT (cyan) and rare Cap2 escapers lack tdT expression (yellow). (**D**) RNAscope and immunostained lung sections showing the expression levels of *Cdkn1a* in the hyperoxia control and $p53^{\Delta EC}$. The $p53^{\Delta EC}$ lung exhibited a significant reduction in *Cdkn1a*-expressing ECs (ERG, yellow), but non-EC *Cdkn1a* expression (cyan) was conserved. (**E**) En-face view of immunostained lungs showing expression of γH2AX in the hyperoxia control and $p53^{\Delta EC}$, showing similar levels of DNA damage in the hyperoxia $p53^{\Delta EC}$ compared to control. (**F**) Violin plot showing expression levels of *Cdkn1a* across cell populations from each cell lineage, showing widespread upregulation of *Cdkn1a* in hyperoxia in nearly every population. (**G**) En-face view of immunostained lungs showing the deletion of *p53* in the epithelium using the Shh^{Cre} driver, which demonstrates no vascular phenotype in room air or hyperoxia assessed by CAR4, PLVAP, and ERG staining. Images are representative of at least 3 littermate pairs. P, postnatal. AT1, alveolar type 1. AT2, alveolar type 2. EC, endothelial cell. myofb, myofibroblast. FB, fibroblast. IM, interstitial macrophage. m, macrophage. Scale bars, 10 µm.

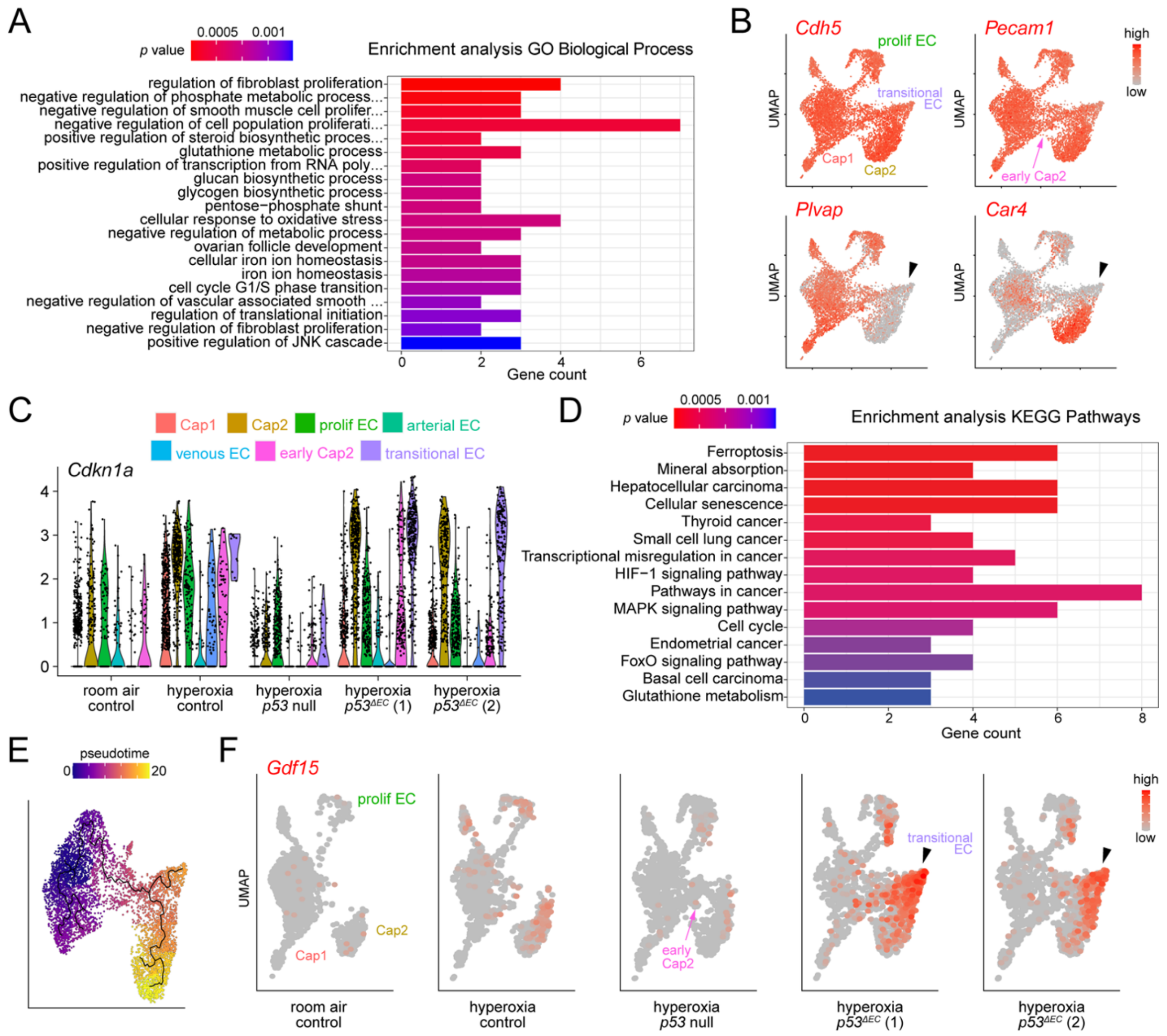

Supplemental Figure 8

(**A**) Gene ontology analysis showing the upregulated biological processes in transitional ECs indicating a role in the response to oxidative and metabolic stress, along with the regulation of other cell populations. (**B**) UMAPs of purified hyperoxia *p53*$^{\Delta EC}$ lung ECs showing expression of EC markers *Cdh5* and *Pecam1*, and EC-type specific markers *Plvap* and *Car4*. Notably, transitional ECs (arrowhead) express both lineage endothelial markers, but lack expression of *Plvap* and *Car4*. Early Cap2 cells express *Plvap* but not *Car4*. (**C**) Violin plots showing expression levels of *Cdkn1a* across EC populations in each condition. *p53*$^{\Delta EC}$ lungs in hyperoxia exhibit substantial expression of *Cdkn1a* in both Cap2 ECs and transitional ECs at RNA level compared to hyperoxia control. (**D**) KEGG pathway enrichment analysis showing an upregulation of senescence related genes in transitional ECs. (**E**) Pseudotime plot showing the trajectory of ECs following the black line. Beginning in the dark blue/purple region, these are the initial cells, while the yellow region represents cells at the end of their trajectory. (**F**) UMAP of lung ECs showing the expression of *Gdf15* across conditions, and its specific upregulation in transitional ECs (arrowhead).

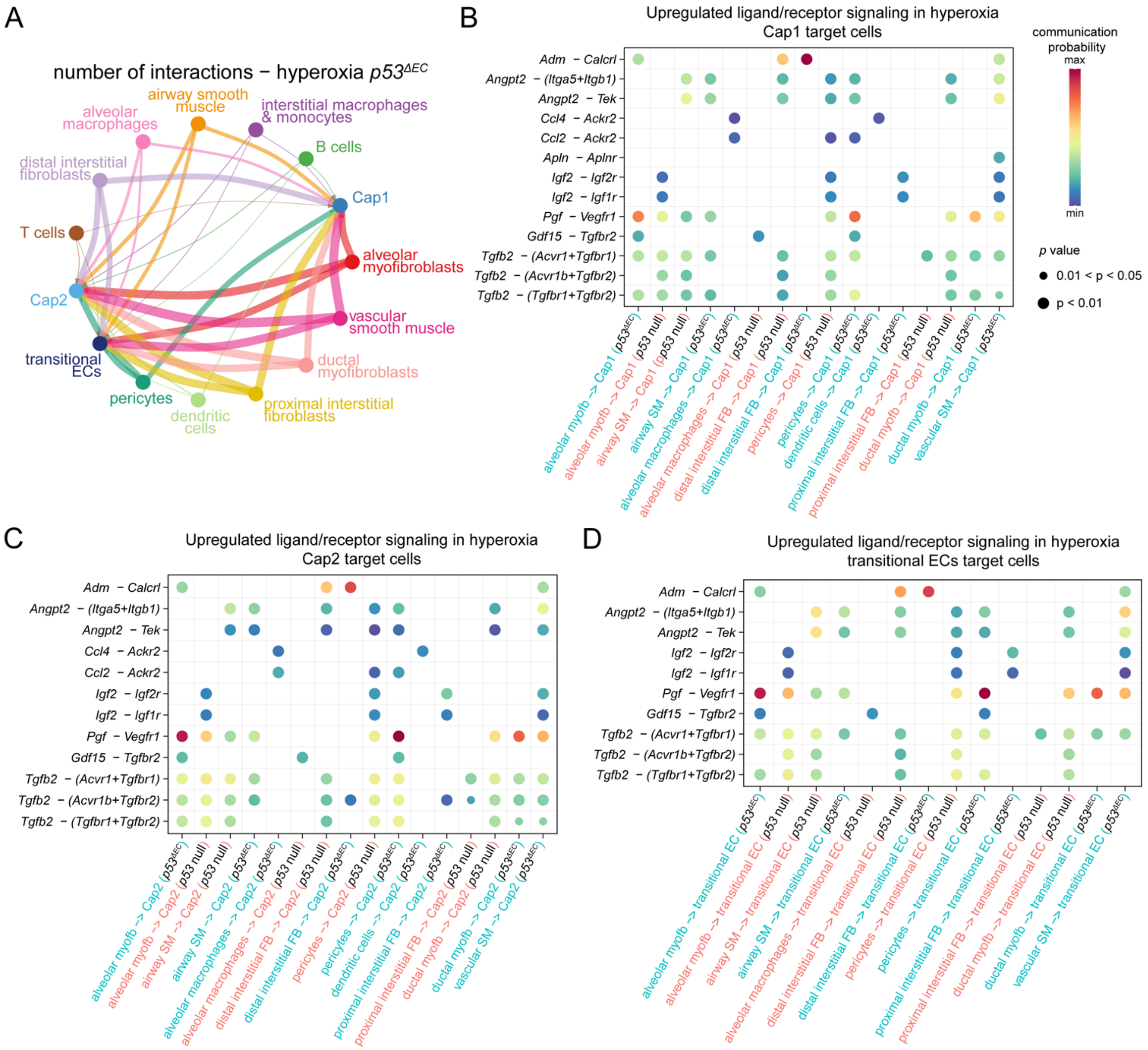

Supplemental Figure 9

(**A**) Circle plot showing ligand-receptor interactions in the hyperoxia *p53*$^{\Delta EC}$ lung with Cap1, Cap2, or transitional ECs as targets. Only mesenchymal and immune interactions with these EC clusters are shown. (**B-D**) DotPlots of predicted ligand-receptor interactions that are upregulated in the *p53*$^{\Delta EC}$ lung ECs. Using Cap1 (**B**), Cap2 (**C**) and transitional ECs (**D**) as targets, we compared potential communications between mesenchymal and immune populations in the *p53*$^{\Delta EC}$ or *p53* null. Although most of the interactions were shared between ECs, differences between mutants are present which could possibly explain the endothelial differences observed in both models. Myofb, myofibroblast. EC, endothelial cell. SM, smooth muscle. FB, fibroblast.

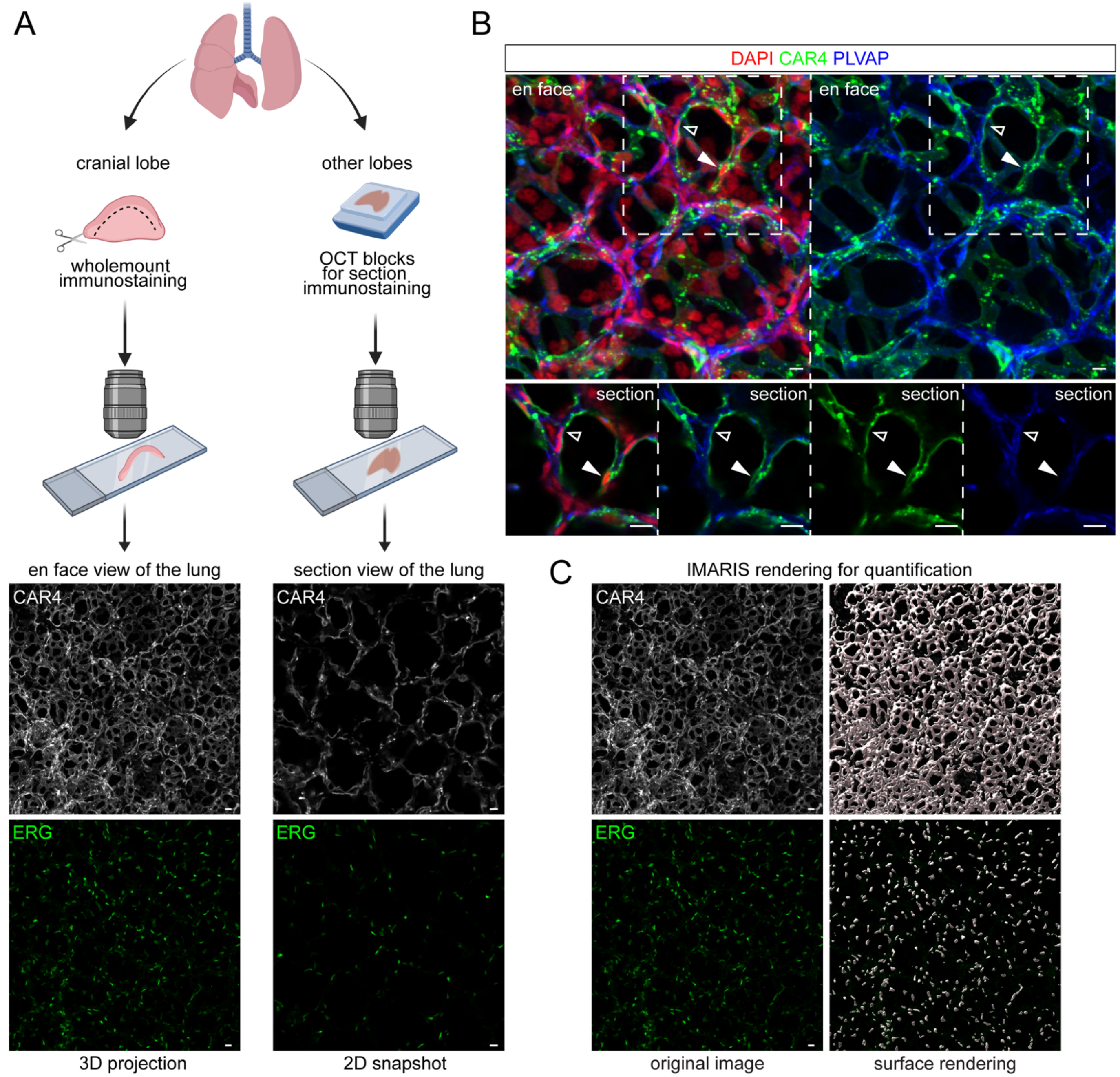

Supplemental Figure 10

(**A**) Experimental model showing the two different methods used for immunostaining. On the left is wholemount immunostaining in which strips are cut from the distal edge of the cranial lobe, stained with antibody and mounted on a slide flat-side up. When imaged on a confocal microscope, a sequence of images is taken at different focal depths, creating a 3D projection of the tissue that gives an en-face view of the lung. On the right is section immunostaining in which the left, caudal, middle, and accessory lobes are frozen in OCT blocks and cut into 20 μm

sections on slides that are then stained and mounted. When imaged on a confocal microscope, a 2D snapshot of the tissue sample is taken, giving a section view of the lung. Created with BioRender. (**B**) En-face and section views of the lung from a wholemount immunostain. The en-face images display a complete depth of 20 μm (projection). To get section views, optical sectioning on the 3D projection reveals a single 1 μm thick slice. This allows for better visualization of co-localization within individual cells as there is no overlap with cells at different focal depths along the Z-axis of the tissue sample. (**C**) IMARIS rendering used for quantification of Cap2 vasculature and endothelial cell number. On the left is the original image and on the right is the surface rendering created by the IMARIS software allowing for image analysis. This method was used for quantification of membrane (top) and nuclear (bottom) proteins. Scale bars, 10 μm.
