# Supplementary figures and images for: p53 maintains lineage fidelity during lung capillary injury-repair in neonatal hyperoxia
Source: JCI Insight. 2025 Aug 5;10(17):e182880. doi: 10.1172/jci.insight.182880 (PMC12487676; doi:10.1172/jci.insight.182880)

Full unedited blots for Supplemental Figure 3C

P53

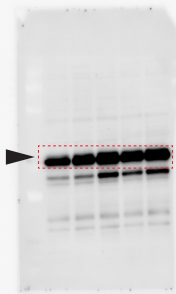

phospho-P53

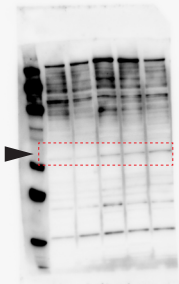

GAPDH

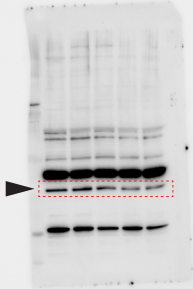

Supplement: Unedited blot and gel images [file jciinsight-10-182880-s207.pdf]
